# Ocular manifestations of the genetic causes of focal and segmental glomerulosclerosis

**DOI:** 10.1007/s00467-023-06073-y

**Published:** 2023-08-14

**Authors:** Victor Zhu, Tess Huang, David Wang, Deb Colville, Heather Mack, Judy Savige

**Affiliations:** 1 Department of Medicine (Melbourne Health and Northern Health), Royal Melbourne Hospital, The University of Melbourne, Parkville, VIC 3050 Australia; 2 Department of Surgery, Royal Victorian Eye and Ear Hospital, The University of Melbourne, East Melbourne, VIC 3002 Australia

**Keywords:** Alport syndrome, Focal and segmental glomerulosclerosis, FSGS, SRNS, Steroid-resistant nephrotic syndrome, Ocular, Inherited retinal dystrophy

## Abstract

Genetic forms of focal and segmental glomerulosclerosis (FSGS) often have extra-renal manifestations. This study examined FSGS-associated genes from the Genomics England Renal proteinuria panel for reported and likely ocular features. Thirty-two of the 55 genes (58%) were associated with ocular abnormalities in human disease, and a further 12 (22%) were expressed in the retina or had an eye phenotype in mouse models. The commonest genes affected in congenital nephrotic syndrome (*NPHS1*, *NPHS2*, *WT1*, *LAMB2*, *PAX2* but not *PLCE1*) may have ocular manifestations . Many genes affected in childhood–adolescent onset FSGS (*NPHS1*, *NPHS2*, *WT1*, *LAMB2*, *SMARCAL1*, *NUP107* but not *TRPC6* or *PLCE1*) have ocular features. The commonest genes affected in adult-onset FSGS (*COL4A3–COL4A5,* *GLA* ) have ocular abnormalities but not the other frequently affected genes (*ACTN4*, *CD2AP*, *INF2*, *TRPC6*). Common ocular associations of genetic FSGS include cataract, myopia, strabismus, ptosis and retinal atrophy. Mitochondrial forms of FSGS (MELAS, MIDD, Kearn’s Sayre disease) are associated with retinal atrophy and inherited retinal degeneration. Some genetic kidney diseases (CAKUT, ciliopathies, tubulopathies) that result in secondary forms of FSGS also have ocular features. Ocular manifestations suggest a genetic basis for FSGS, often help identify the affected gene, and prompt genetic testing. In general, ocular abnormalities require early evaluation by an ophthalmologist, and sometimes, monitoring or treatment to improve vision or prevent visual loss from complications. In addition, the patient should be examined for other syndromic features and first degree family members assessed.

**Supplementary Information:**

The online version contains supplementary material available at 10.1007/s00467-023-06073-y.

## Introduction

Focal and segmental glomerulosclerosis (FSGS) is a histopathological diagnosis characterised by sclerosis of less than half the glomerular tuft in fewer than half the glomeruli in a kidney biopsy [1].

FSGS is commonly classified into primary, secondary or genetic forms. Primary autoimmune FSGS typically presents with the nephrotic syndrome (NS), responds to immunosuppression (‘steroid-sensitive’ , SSNS) and recurs after transplantation [2, 3]. The genetic forms of FSGS frequently manifest with lower levels of proteinuria [4], and neither respond to steroid treatment (‘steroid-resistant’ nephrotic syndrome, SRNS) nor recur post transplantation [5]. Secondary FSGS results from hyperfiltration after nephron loss. Each disease type has implications for management and prognosis.

An accurate and timely diagnosis is critical in providing effective treatment, advising other family members of the risk of being affected and in avoiding the complications of steroid treatment. However, identification of individual genetic forms of FSGS is generally not possible with biopsy alone and requires genetic testing [6].

There are however clues to the likelihood of a genetic basis for FSGS [7]. These include a positive family history [8], a young age at onset [9], SRNS [10] and no other obvious cause [11]. The association with extra-renal features, such as a hearing loss, skeletal, cardiac or ocular anomalies, is also important [12]. 

More than 70 genes have been implicated in FSGS, and inheritance is mainly autosomal recessive (AR) in children but often autosomal dominant (AD) in adults, as well as X-linked (XL) or mitochondrial [13]. The commonest genes associated with nephrotic syndrome differ at birth (*NPHS1*, *NPHS2*, *WT1*, *LAMB2*, *PAX2*, *PLCE1*), in childhood or adolescence (*NPHS1*, *NPHS2*, *WT1*, *LAMB2*, *SMARCAL1*, *NUP107*, *TRPC6*, *PLCE1*) and in adults (*COL4A3*-*COL4A5*, *GLA*, *ACTN4*, *CD2AP*, *INF2*, *TRPC6*).

Overall, the commonest genes affected in FSGS are *COL4A5* (XL Alport syndrome) and *COL4A3* and *COL4A4* (usually AD rather than the rare AR Alport syndrome) *INF2*, *TRPC6* and *ACTN4* [8, 14, 15]. Most of these genes code for proteins that are found in the glomerular podocyte or adjacent extracellular matrix. There is also overlap with genes that result in other kidney phenotypes including some forms of congenital abnormalities of the kidney and urinary tract (CAKUT), cystic kidney diseases, renal ciliopathies and tubulopathies [13, 16]. These include Dent disease (*CLCN5*, *OCRL*), AD tubulointerstitial kidney disease (ADTKD due to *UMOD* variants), nephronophthisis (*TTC21B*, *NPHP4*), Imerslund-Grasbeck syndrome 1 (*CUBN*) and papillorenal syndrome (*PAX2*). Finally, some mitochondrial diseases (mitochondrial encephalopathy, lactic acidosis and stroke-like episodes (MELAS) [17], maternally inherited diabetes and deafness (MIDD) [18], and Kearns-Sayre syndrome [19]) are associated with FSGS.

Some of these diseases are suspected on the basis of their extra-renal features. Ocular abnormalities are particularly common in genetic kidney disease and while these may not severely affect vision, they are helpful indicators of the genetic nature of the underlying disease [20, 21]. The association between kidney and eye disease is attributable to developmental, structural and physiological similarities between the kidney and the eye [22]. Both the kidney and the eye are ‘paired’ organs that share some transcription factors; and the glomerular filtration barrier resembles the retina [23] with epithelial cells overlying a basement membrane of mainly collagen IV α3α4α5 and a capillary endothelium [23]. Other similarities occur in the microcirculation with a localised renin–angiotensin system in both the glomerular and retinal vasculature [24].

The presence of ophthalmological abnormalities in a person with FSGS suggests a genetic basis and encourages genetic testing. It also facilitates early ophthalmological evaluation and monitoring to prevent vision loss. It may also provide insights into genetic kidney disease pathogenesis.

This review characterises the ocular associations of the individual genes affected in FSGS that may be useful in indicating an underlying genetic disorder and, in some cases, the specific gene affected.

## Methods

The genes for FSGS from the Renal Proteinuria panel were down-loaded from the Genomics England Panel App in October 2020 (v2.77, green and amber genes).

Genomics England uses a traffic light system where ‘green’ genes have a high level of evidence for an association with a disease (having been reported in 3 unrelated families or 2 families with further strong evidence) as decided by an expert panel. These represent the genes that should be examined in a diagnostic genetic laboratory. ‘Amber’ genes have borderline levels of evidence and 'red' genes have low levels for a disease association.

Genes associated with FSGS were then searched in Medline (OVID), Embase and the Cochrane Database of Systematic Reviews with the terms (eye* or ocular or retina* or lens or cornea* or vision or ophthalm*) and ‘gene name’ to identify all reports of ocular manifestations. All manuscripts in English likely to include ocular manifestations of FSGS based on their abstracts were read. Full-text articles that did not report FSGS or ocular findings, and those where only the abstract was available or were a conference proceeding were excluded. Additional references from studies were hand- searched. In addition, Online Mendelian Inheritance in Man (OMIM: https://www.omim.org/) was used to identify renal, extra-renal and further ocular features in October 2022.

Since some genes were reported in only a few individuals who had not necessarily undergone a complete ophthalmological examination, two further databases were studied to determine whether ophthalmic features were likely. These were the Human Protein Atlas (https://www.proteinatlas.org/) which was examined for mRNA expression in the retina and the Mouse Genome Informatics database (http://www.informatics.jax.org/) which was examined for an ocular phenotype in mouse models. Searches were undertaken August–September 2020 and reviewed October 2022.

In addition to the Renal Proteinuria panel, the genes associated with secondary FSGS (*CLCN5*, *OCRL*, *CUBN*, *PAX2*) and the common FSGS-associated mitochondrial diseases (MELAS, MIDD, Kearns Sayre disease) were searched.

## Results

**Fig. 1:**
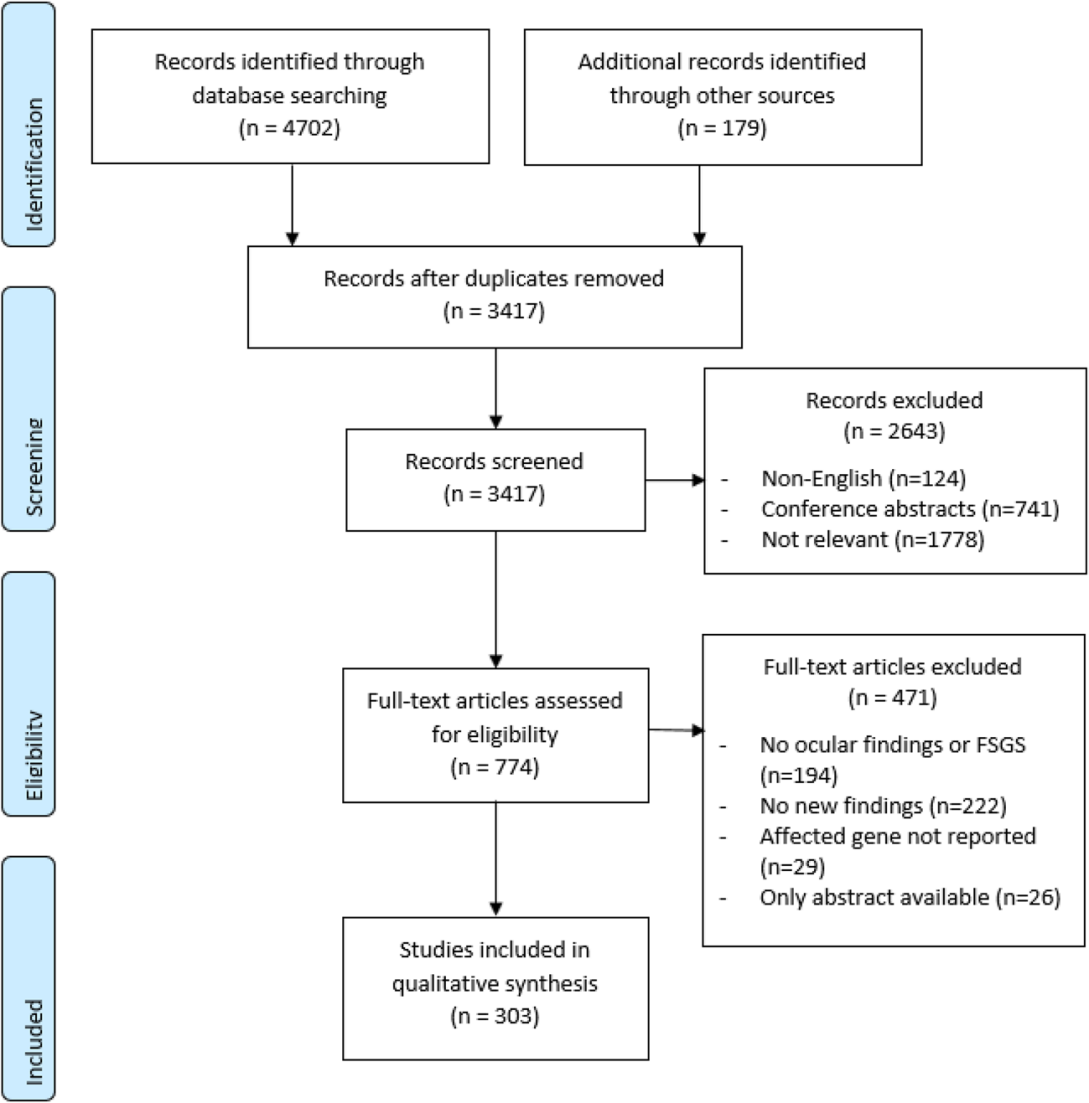
PRISMA flow diagram for the selection of studies

In all, 4702 records were identified from the databases and a further 179 from hand searching. After duplicates were removed, 3417 abstracts were screened to yield 774 full texts, and after review, 303 records were examined (Fig. 1).

**Fig. 2:**
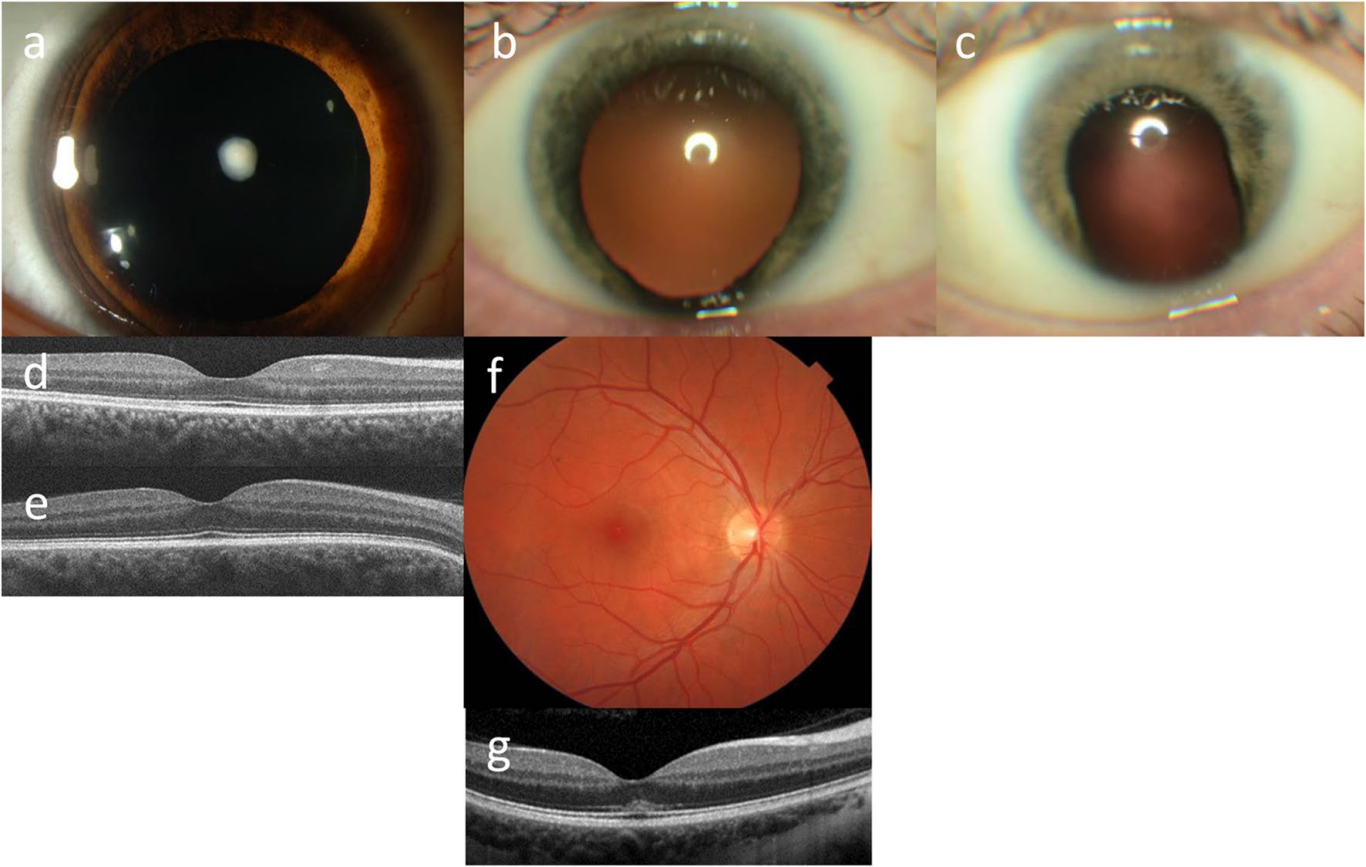
Ocular features associated with genetic causes of FSGS demonstrating **a** congenital cataract; **b** inferior coloboma; **c** microphthalmia with inferior coloboma in partner eye of (**b**); **d** retinal profile on optical coherence tomography in Pierson syndrome presenting in adulthood; **e** retinal profile in X-linked Alport syndrome demonstrating the resemblance; **f** cherry red spot macula typical of many metabolic diseases including Fabry disease; and **g** optical coherence tomography profile of the retina with accumulation at the level of the external limiting membrane

**Fig. 3:**
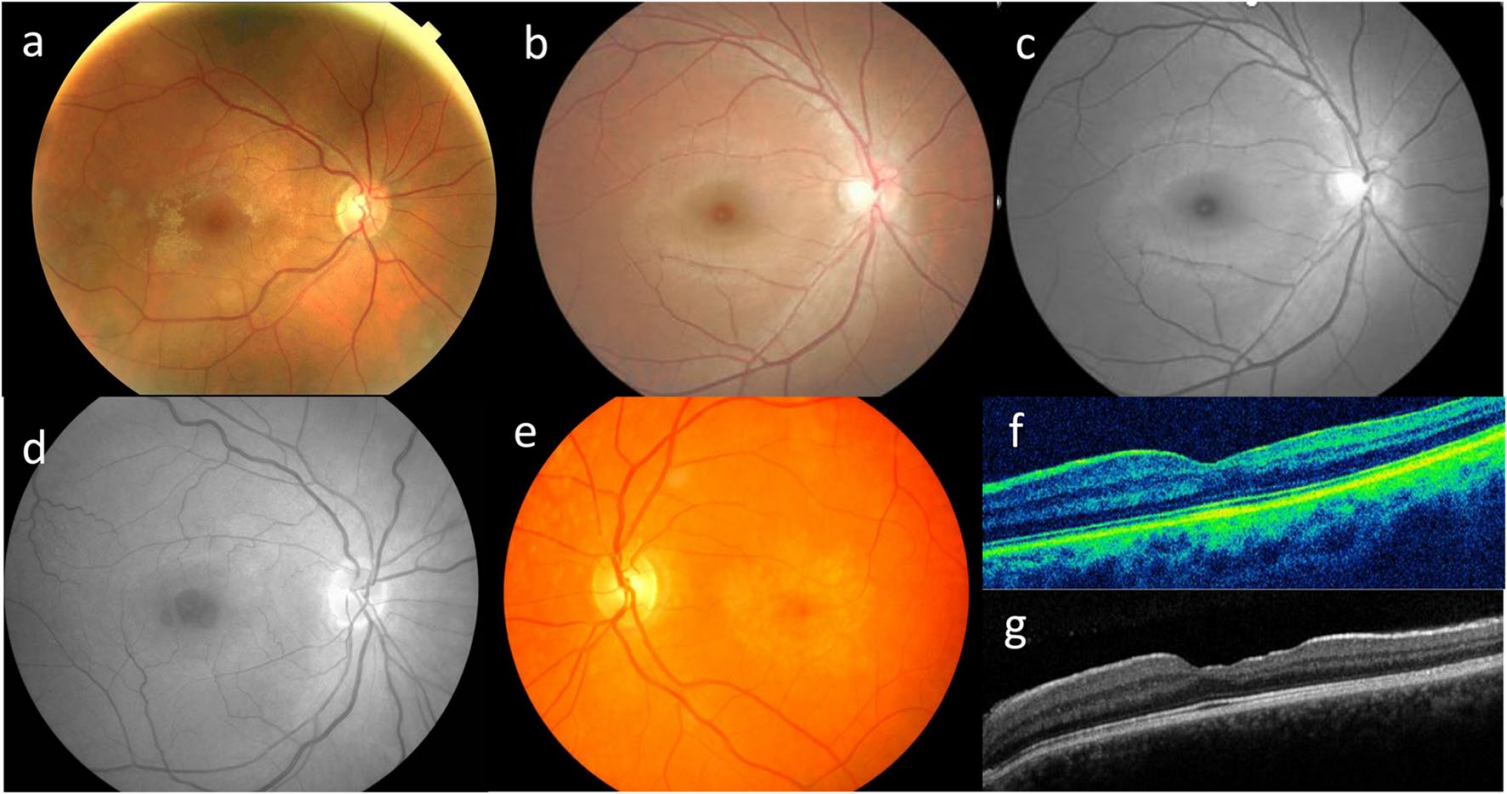
Ocular features associated with XL or AR Alport syndrome demonstrating **a** perimacular fleck retinopathy; **b** subtle perimacular fleck retinopathy that must be distinguished from the normal retinal sheen seen in young people; **c** black and white image of (**b**) demonstrating the fleck retinopathy more clearly; **d** black and white image demonstrating the margins of a large macular hole; **e** maculopathy; **f** typical profile on optical coherence tomography demonstrating the > 10% temporal retinal atrophy; and **g** retinal profile on optical coherence tomography demonstrating a lamellar macular hole

**Fig. 4:**
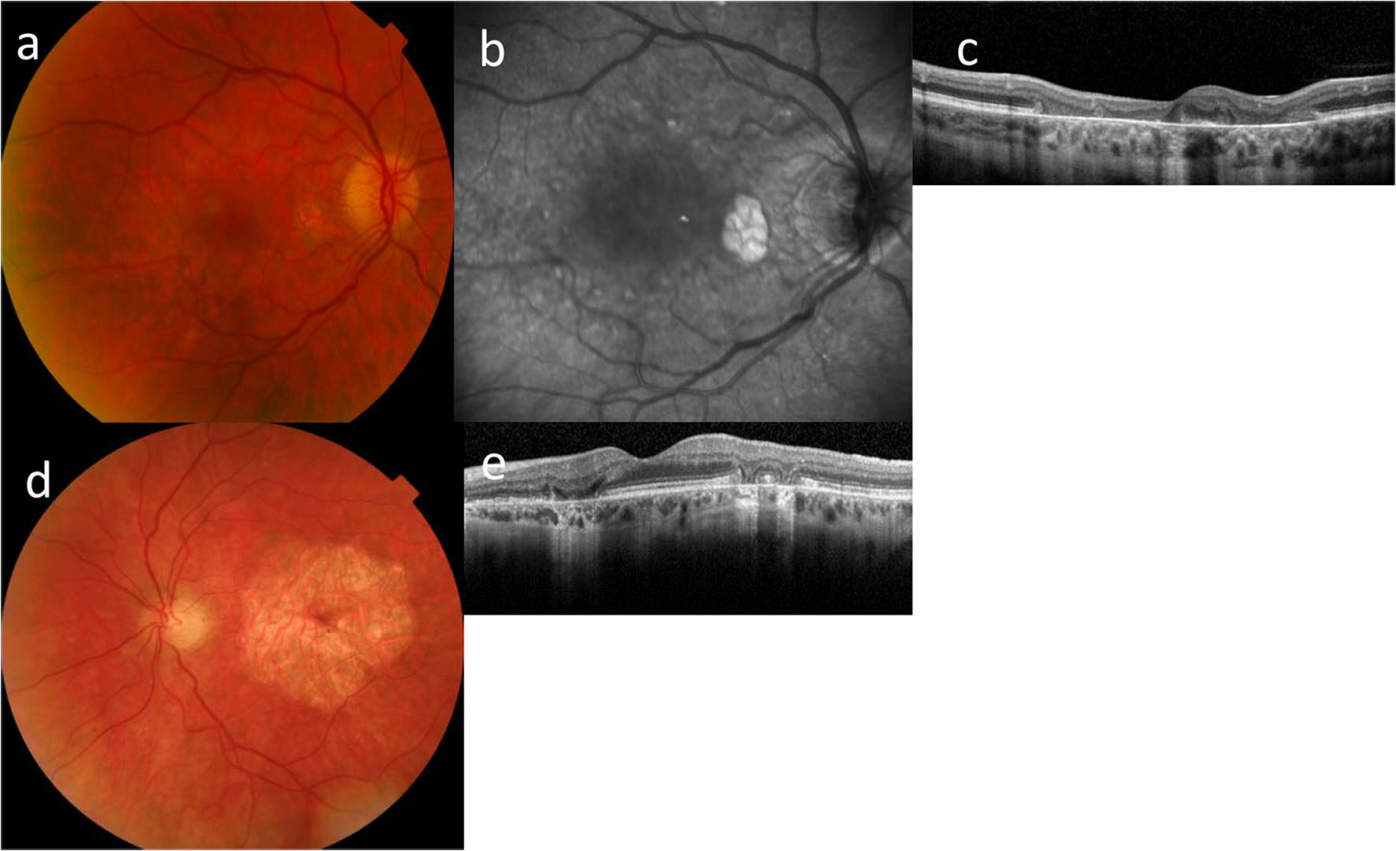
Ocular features associated with FSGS due to mitochondrial diseases demonstrating **a** MELAS with focal atrophy in the right eye and generalised perifoveal thinning; **b** red-free photograph with more extensive areas of foveal atrophy; **c** optical coherence tomography scan with areas of atrophy confirming the foveal defects in the outer retina and retinal pigment epithelium; **d** MIDD demonstrating normal fovea surrounded by extensive atrophy; and **e** optical coherence tomography demonstrating parafoveal atrophy of the outer retinal layer

Fifty-five genes from the Genomics England Renal proteinuria panel were studied. Thirty-two (58%) had ocular manifestations reported in human disease (Tables 1, 2 and 3; Figs. 2, 3 and 4).

**Table 1:** Genes associated with FSGS, ocular features, retinal expression, and ocular phenotypes in mouse models

Gene (OMIM)	Disease (OMIM)	Function	Kidney features	Extrarenal features (OMIM)	Ocular features	Retinal mRNA expression in Human Protein Atlas	Ocular phenotype in mouse model
*ACTN4* (604638) (GE- green)	FSGS-1 (603278) AD	Cytoskeleton	FSGS	None reported	Retinal venular tortuosity (GWAS)	26.0 TPM	Abnormal eye muscle, lens, optic cup, retina
*AMN* (605799) (GE- amber)	Imerslund–Grasbeck syndrome 2 (618882) AR	Trans-membrane protein	Mild proteinuria	Malabsorption of vitamin B12, megaloblastic anaemia	Anterior polar cataract, posterior subcapsular cataract	0 TPM	Microphthalmia
*ANLN* (616027) (GE- amber)	FSGS-6 (616032) AD	Cytoskeleton	FSGS	None reported	None reported	0.3 TPM	No eye pathology noted
*ARHGDIA* (*601925*) (GE- green)	Nephrotic syndrome type 8 (615244 (AR)	Rho-GDP dissociation inhibitor	Proteinuria, FSGS	Hearing loss, intellectual disability	None reported	14.6 TPM	No eye pathology noted
*CD151 (602243)* (GE- amber)	Epidermolysis bullosa simplex 7, with nephropathy and deafness (609057) AR	Trans-membrane protein	Kidney agenesis, proteinuria, nephrotic syndrome	Hearing loss, pretibial bullae	Bilateral lacrimal duct stenosis	9.6 TPM	No eye pathology noted
*CD2AP* (604241) (GE-amber)	FSGS-3 (607832) AR	Slit diaphragm complex	FSGS	None reported	None reported	6.9 TPM	No eye pathology noted
*CLCN5* (*300008*) (GE- green)	Dent disease 1 (300009); Proteinuria (308990) XL	Proximal renal tubular defect	Decreased phosphate reabsorption; stones, proteinuria	Rickets, osteomalacia	Cataract	3.4 TPM	No eye pathology noted
*COL4A3* (*120070*) *COL4A4* (*120131*)	Alport syndrome XL, AR	Glomerular basement membrane and receptors, cell matrix	Haematuria, proteinuria, FSGS	Sensorineural hearing loss	Myopia, recurrent corneal erosions, posterior polymorphous corneal dystrophy, corneal arcus, pigment dispersion syndrome, anterior lenticonus, cataract, anterior polar cataract, posterior lenticonus, central fleck retinopathy, peripheral retinopathy, bull’s eye maculopathy, macular holes, temporal retinal thinning, retinal lozenge, vitelliform maculopathy	52.9 TPM; 8.4 TPM; 6.1 TPM	Abnormal lens, abnormal eye physiology; irregularly shaped pupil; cataract
*COL4A5 (303630) (GE- all green)*				(*COL4A5* variants only) Leiomyomata in trachea, bronchi, female genitalia; aortic, cerebral and coronary artery aneurysms			
*COQ2* (609825) (GE- green)	Coenzyme Q10 deficiency, primary, 1 (607426) AR	Mitochondrial function	FSGS, collapsing glomerulopathy crescentic glomerulonephritis	Cerebellar ataxia, myoglobinuria, progressive muscle weakness, lactic acidaemia, encephalopathy, hypotonia, psychomotor delay, seizures, hypertrophic cardiomyopathy	Optic atrophy, Inherited retinal degeneration	4.0 TPM	No eye pathology noted
*COQ6* (614,647) (GE- green)	Coenzyme Q10 deficiency, primary, 6 (614650) AR	Mitochondrial function	FSGS, diffuse mesangial sclerosis	Seizures, ataxia, facial dysmorphism, sensorineural hearing loss	Bilateral optic nerve atrophy, exotropia with nystagmus	5.8 TPM	Abnormal cornea, abnormal vitreous
*COQ8B* (615,567) (GE- green)	Nephrotic syndrome type 9 (615573) (AR)	Mitochondrial function	FSGS, collapsing FSGS	Mild intellectual disability, seizures, thoracic aortic aneurysm	Inherited retinal degeneration	1.3 TPM	No eye pathology noted
*CRB2* (609720) (GE- green)	FSGS-9 (616220) AR	Slit diaphragm complex	FSGS, cysts	Ventriculomegaly, aqueductal stenosis, cerebellar hypoplasia, seizures, quadrigeminal cysts, atrial or ventricular septal defect, scimitar syndrome	Myopia, optic atrophy, pale retina, macular pit	8.1 TPM; high in limiting membrane	Retinal degeneration
*CUBN* (*602997*) (GE- green)	Imerslund–Grasbeck syndrome 1 (261100), AR	Intestinal receptor for Intrinsic factor	Proteinuria	B12 malabsorption, megaloblastic anaemia	None reported	3.0 TPM	Fused cornea and lens, iris synechiae, abnormal iris morphology
*DGKE (601440)* (*GE-amber*)	Nephrotic syndrome type 7 (615008) AR	Intracellular lipid kinase	Nephrotic syndrome, atypical haemolytic uremic syndrome (in some)	Haemolytic anaemia, thrombocytopenia in some patients	None reported	34.5 TPM	No eye pathology noted
*DLC1* (*604258*) (GE- green)	SSNS and SRNS, AR	Rho GTPase	Proteinuria	None reported	None reported	1.5 TPM	No eye pathology noted
*EMP2* (*602,334*) (GE- green)	Nephrotic syndrome type 10 (615861) AR	Tetraspaninin protein	Proteinuria	None reported	None reported	2.0 TPM	No eye pathology noted
*FAT1 (600976)* (GE- green)	Proteinuria	Cadherin-like protein	Glomerulo-tubular nephropathy	None reported	None reported	3.4 TPM	Aniridia, aphakia, microphthalmia, inherited retinal degeneration, abnormal retinal vasculature
*FN1 (135600)* (GE-amber)	Glomerulopathy with fibronectin deposits 2 (601894) AD	Glycoprotein on cell surface and in fluids	Proteinuria, kidney failure	None reported	None reported	6.0 TPM	No eye pathology noted
*GLA (300644)* (GE–amber list)	Fabry disease (301500) XL	Galactosidase	Fabry disease	Angiokeratomas; acroparaesthesia; cardiac hypertrophy; hypohydrosis, mitral valve prolapse; episodic diarrhoea; abdominal pain; cerebro-vascular disease; dysmorphic facial and extremity features	Conjunctival lymphangiectasia, reduced lacrimal secretions, upper lid vessel tortuosity, corkscrew arterioles on conjunctivae, corneal verticillate, fine brown subepithelial corneal lines, anterior capsule lens opacification, wedge-shaped lens opacities, branching spoke-like posterior subcapsular cataract, optic neuritis, corkscrew tortuosity of retinal vessels, retinal capillary micro and macroaneurysms, retinal oedema, central retinal artery occlusion, macular choroidal neovascularisation, blind spot enlargement	6.9 TPM	No eye pathology noted
*INF2* (610982) (GE- green);	Charcot–Marie–Tooth disease,	Cytoskeleton, mitochondrial function	FSGS	‘Claw hand’ deformities, symmetrical muscle atrophy, peripheral nerve dysfunction, moderate-severe muscle weakness, sensorineural hearing loss	None reported	3.3 TPM	No eye pathology noted
*INF2 (610982) (GE- green)*	FSGS-5 (613237)						
*ITGA3* (605025) *GAPB3* (GE- green)	Interstitial lung disease, nephrotic syndrome, and epidermolysis bullosa, congenital (614748) AR	Glomerular basement membrane and receptors	FSGS	Skin fragility; blisters; sparse scalp hair, eyebrows and eyelashes; nail dystrophy; distal onycholysis following mild trauma; respiratory distress; diffuse interstitial lung disease; epidermolysis bullosa; mitral insufficiency	None reported	4.4 TPM	No eye pathology noted
*ITSN1* (*602442*) (GE- green)	Steroid-sensitive nephrotic syndrome, AR		Proteinuria	None reported	None reported	28.5 TPM	No eye pathology noted
KANK2 614610 (GE- amber)	Nephrotic syndrome type 16 (617783) AR	Regulate actin polymerisation	Nephrotic syndrome, steroid sensitive	None or with palmoplantar keratoderma and woolly hair (616,099)	None reported	19.0 TPM	Abnormal eye morphology
LAGE3 (300060) (GE- green)	Galloway–Mowat syndrome (301006) XL	Not known	Nephrotic syndrome, SSNS, kidney failure	Microcephaly, intellectual disability, seizures	Nystagmus	4.6 TPM	No eye pathology noted
*LAMA5 (601033)* (GE – amber)	Nephrotic syndrome 26 (620049) AR	Component of glomerular basement membrane	Nephrotic syndrome, kidney failure	Scarring of the skin, muscle weakness, ligamentous laxity, malabsorption and hypothyroidism	Night blindness	0.6 TPM	Microphthalmia, anophthalmia
*LAMB2* (150325) (GE- green);	Nephrotic syndrome type 5 (614199) AR	GBM and receptors, cell matrix	FSGS	Delayed motor milestones, severe proximal limb muscle weakness, scoliosis	Ptosis, myopia, hypopigmented retina, hypoplastic macular areas, poor foveal reflex, small pupils, limited external ocular movements, nystagmus, strabismus	11.7 TPM	Abnormal retina
*LAMB2 (150325) (GE- green)*	Pierson syndrome (609049) AR	GBM and receptors, cell matrix	FSGS, diffuse mesangial sclerosis, minimal change disease, membranous glomerulo-nephritis	Neurodevelopmental delay, hypotonia, progressive microcephaly, scoliosis, genu varum	Microphthalmia, high myopia, posterior staphyloma, microcornea, megalocornea, clouded cornea, band keratopathy, anterior segment dysgenesis, shallow anterior chamber, iris hypoplasia, enlarged iris vessels, posterior synechiae, ectropion uvea, remnants of pupillary membranes, spherophakia, posterior lenticonus, cataract, microphakia, hypoplastic optic nerve, hypopigmented fundus, macular hypoplasia, retinal detachment, tortuous retinal vessels, inherited retinal degeneration, vitreoretinal adhesions and traction, atrophic choriocapillaris, microcoria, nystagmus, strabismus		
*LCAT* (606967) (GE- amber)	Norum disease (245900) AR	Enzyme	Proteinuria, kidney failure	Haemolytic anaemia	Corneal lipid deposits, corneal opacities	2.8 TPM	No eye pathology noted
*LMX1B* (602575) (GE- green)	Nail-patella syndrome (161200) AD	DNA repair, transcription, nuclear transport	Diffuse hyaline thickening of GBM, FSGS, FSGS without extrarenal involvement	Nail anomalies, triangular lunulae, swan neck deformity of fingers, absent creases on dorsal distal interphalangeal joints of fingers, scoliosis, patellofemoral joint anomalies, elbow anomalies, pelvis anomalies, sensorineural hearing loss; weak, crumbling teeth; epilepsy hypertelorism, epicanthal folds	Ptosis, microcornea, sclerocornea, keratoconus, corneal oedema, microphakia, congenital cataract, cloverleaf hyperpigmentation around the central iris (Lester’s sign), anterior synechiae, glaucoma, ocular hypertension, multiple iris processes, heterochromia, pigment dispersion syndrome, anisocoria, strabismus	0 TPM	Abnormal iris, abnormal cornea, corneal ulcers, abnormal lens, microphthalmia
*MAGI2* (606382) (GE- green)	Nephrotic syndrome type 15 (61,609)	Cell junction organisation	Nephrotic syndrome	None reported	None reported	11.6 TPM	No eye pathology noted
*MYH9* (160775) (GE- green)	Macrothrombo-cytopenia, granulocyte inclusions with or without nephritis or sensorineural hearing loss (155100) AD	Cytoskeleton	FSGS	Thrombocytopaenia, giant platelets, high frequency sensorineural hearing loss	Congenital cataract, presenile cataract, neovascularisation, retinal haemorrhage, vitreous haemorrhage, peripheral retinal pigmentary changes	9.7 TPM	Abnormal cornea, abnormal lens
*MYO1E* (601479) (GE- green)	FSGS-6 (614,131) AR	Slit diaphragm complex, cytoskeleton	FSGS	None reported	None reported	9.9 TPM	No eye pathology noted
*NPHS1* (602716) (GE- green)	Nephrotic syndrome type 1 (256300) AR	Slit diaphragm complex	FSGS, minimal change glomerulo-nephritis	Pyloric stenosis	Myopia, high hyperopia, bilateral posterior subcapsular cataract, strabismus, amblyopia	0.7 TPM	No eye pathology noted
*NPHS2* (604766) (GE- green)	Nephrotic syndrome type 2 (600995) AR	Slit diaphragm complex	FSGS	None reported	Myopic astigmatism, cataract, anisometropic amblyopia, exotropia	0 TPM	No eye pathology noted
*NUP85* (170285) *NUP75* (GE- green)	Nephrotic syndrome type 17 (618176) AR	Nuclear proteins and transcription factors	FSGS	Intellectual disability, short stature	None reported	11.2 TPM	No eye pathology noted
*NUP93* (614351) (GE- green)	Nephrotic syndrome type 12 (616892) AR	Nuclear proteins and transcription factors	FSGS diffuse mesangial sclerosis	None reported	None reported	31.7 TPM	No eye pathology noted
*NUP107* (607617) (GE-green)	Galloway–Mowat syndrome 7 (618348) AR; Nephrotic syndrome, type 11 (616730) AR	Nuclear proteins and transcription factors	FSGS, IgA nephropathy; FSGS, minimal change nephrotic syndrome, diffuse mesangial sclerosis	Mild-moderate intellectual disability, developmental delay, microcephaly, sloping forehead, bitemporal narrowing, smooth philtrum, micrognathia, simian crease, clinodactyly, right brachial plexopathy, bifid thumb, cubitus valgus, hallux valgus, pectus excavatum, kyphoscoliosis, short stature; cleft lip, cleft palate	Hypertelorism (OMIM)	14.7 TPM	No eye pathology noted
*NUP133* (607613) (GE-green)	Galloway–Mowat syndrome 8 (618349) AR	Nuclear proteins and transcription factors	FSGS	Psychomotor retardation, hypotonia, microcephaly, bilateral thumb deviation, enamel hypoplasia, atopic dermatitis, epilepsy, talipes calcaneus, equinus foot	Convergent strabismus	19.1 TPM	No eye pathology noted
*OCRL* (*300535*) (GE- green)	Dent disease 2; Lowe syndrome, XL	Membrane trafficking	Proximal tubular defect; amino-aciduria; nephron-calcinosis, kidney failure	Developmental delay, intellectual impairment	Cataracts present at birth, glaucoma, microphthalmia, impaired visual acuity, (Lowe syndrome)	9.9 TPM	Abnormal eye
*OSGEP* (*610107*) (GE-green)	Galloway–Mowat syndrome 3 (617729) AR		Nephrotic syndrome, kidney failure	Microcephaly, micrognathia, arachnodactyly, intellectual disability	Microphthalmia, strabismus, hypertelorism, impaired vision	4.1 TPM	No eye pathology noted
*PAX2* (167409) (GE-green)	FSGS-7 (616002) AD	DNA repair, transcription, nuclear transport	Adult onset FSGS	Mild hypertrophic cardiomyopathy, microcephaly, cryptorchidism	Optic nerve coloboma; optic disc coloboma, optic disc pit, optic nerve atrophy, glaucomatous cupping	1.2 TPM	Coloboma, abnormal retina, abnormal blood vessels
*PDSS2* (610564) (GE- green)	Coenzyme Q10 Deficiency, Primary, 3 (614652) AR	Mitochondrial function	Susceptibility to FSGS	Hypotonia, seizures	Cortical blindness, Leigh syndrome	6.0 TPM	No eye pathology noted
*PLCE1* (608414) (GE- green)	Nephrotic syndrome type 3 (610725) AR	Cell signalling slit diaphragm	FSGS, diffuse mesangial sclerosis	None reported	Glaucoma	7.3 TPM	No eye pathology noted
*PODXL* (602632) (GE- green)	AR	Cell membrane associated protein	FSGS	None reported	None reported	67.8 TPM	Abnormal retinal pathology
*PTPRO* 600579 (GE-amber)	Nephrotic syndrome type 6 (614196) AR	Podocyte tyrosine phosphatase	FSGS	None reported	None reported	7.5 TPM	No eye pathology noted
*SCARB2* (602257) (GE- green)	Epilepsy, progressive myoclonic 4, with or without kidney failure (254900) AR	Lysosome	FSGS	Progressive fine tremor, action myoclonus, convulsive seizures, ataxia, dysarthria, slight cognitive impairment, rapid eye movement sleep disorder	Slowed horizontal saccades	53.2 TPM	No eye pathology noted
*SGPL1* (603729) (GE-green)	Nephrotic syndrome type 14 (617575) AR	Metabolic and cytosolic	FSGS, diffuse mesangial sclerosis	Primary adrenal insufficiency, hyperpigmentation, ichthyosis, primary hypothyroidism, neurodevelopmental delay, ataxia, cognitive decline, sensorineural deafness, seizures, lymphopaenia, hypocalcaemia, hypoglycaemia, dyslipidaemia, cryptorchidism, micropenis, microcephaly, hypotonia, peripheral neuropathy	Bilateral cataracts, salt and pepper retinopathy, ptosis, esotropia, amblyopia, strabismus	10.8 TPM	No eye pathology noted
*SMARCAL1* (606622) (GE- green)	Schimke immunoosseous dysplasia (242900) AR	DNA repair, transcription, nuclear transport	FSGS	Lymphopenia. multiple lentigines, spondyloepiphyseal dysplasia, cerebral infarcts	Astigmatism, corneal opacity, myopia	5.8 TPM	No eye pathology noted
*TBC1D8B* (301027) (GE- green)	Nephrotic syndrome type 20 (301028) XL	Metabolic and cytosolic	FSGS, kidney failure	None reported	None reported	9.6 TPM	No eye pathology noted
*TPRKB (*608680) (GE–amber)	Galloway–Mowat syndrome 5 (617731) AR	Part of KEOPS complex	FSGS, kidney failure	Microcephaly, delayed psychomotor development, demyelination	Hypertelorism, epicanthal folds	16.1 TPM	No eye pathology noted
*TNS2* (*607717*) (GE- green)	Nephrotic syndrome type 22	Binds to actin	Minimal change diffuse mesangial sclerosis	None reported	None reported	6.0 TPM	No eye pathology noted
*TP53RK* (608679) (GE- green)	Galloway–Mowat syndrome 4 (617730) AR	DNA repair, transcription	FSGS, diffuse mesangial sclerosis	Primary microcephaly, seizures, developmental delay, cognitive impairment, hypotonia, spasticity, facial dysmorphism, short stature, tapered fingers, feeding difficulties, multiple hypo- and hyperpigmented macules	Hypertelorism, visual impairment	6.4 TPM	No eye pathology noted
*TRPC6* (603652) (GE- green)	FSGS-2 (603965) AD	Cell signalling, slit diaphragm	FSGS, minimal change disease, kidney failure	None reported	None reported	0.3 TPM	Abnormal eye physiology
*WDR73* (616144) (GE- green)	Galloway–Mowat syndrome 1 (251300) AR	DNA repair, transcription, nuclear transport	FSGS diffuse mesangial sclerosis	Microcephaly, hiatus hernia, hypotonia, abnormal skull, flat occiput, seizures, developmental delay, bilateral club feet, micrognathia, close-set eyes, large ears, small midface, aqueductal stenosis, epicanthal folds, broad nasal bridge, hypertelorism, congenital hypothyroidism, contractures, bilateral simian crease, thyroid dysplasia, adrenal hypoplasia, hypertonia, spastic quadriplegia, short stature, dystonia, ataxia, hirsutism, hip dysplasia	Myopia, optic atrophy, optic neuropathy, retinopathy, abnormal visual evoked potentials, strabismus, nystagmus, oculomotor apraxia	3.4 TPM	No eye pathology noted
*WT1* (607102) (GE- green)	*WT1* diseases, FSGS-4 (256370) AD	DNA repair, transcription, nuclear transport	Wilms tumour FSGS-4, diffuse mesangial sclerosis	Congenital hemihypertrophy, genitourinary abnormalities,intellectual disability, diaphragmatic hernia, hypomyelinating leukodystrophy	Bilateral ptosis, hazy cornea, nuclear and foetal cataracts, anterior polar cataracts, posterior subcapsular cataracts, increased intraocular pressure, aniridia, bilateral optic atrophy	0 TPM	No eye pathology noted

References for the ocular features are provided in the Supplementary file

*AD* autosomal dominant, *AR* autosomal recessive, XL X-linked, *CAKUT* congenital anomalies of the kidney and urinary tract, *FSGS* focal segmental glomerulosclerosis. *GE-*, gene is from the Genomics England panel for Renal proteinuria. These panels use a traffic light system where ‘green’ genes have the best evidence for an association with the disease (pathogenic variants have been reported in at least 3 families or two families with other evidence) or ‘amber’ where the evidence is less than this as assessed by an expert panel

**Table 2:** Common mitochondrial diseases causing FSGS, ocular features, retinal expression and ocular phenotypes in mouse models

Disease (OMIM)	Genes (OMIM)	Kidney features	Extrarenal features	Ocular features
Myopathy, Encephalopathy, Lactic Acidosis, Stroke (MELAS) syndrome	*MT-TL1* and other genes	FSGS, tubulointerstitial nephropathy, kidney failure	Stroke, seizures, lactic acidosis, dementia, short stature, hearing loss, learning disability, myoclonus, cerebellar signs, congestive heart failure, Wolff–Parkinson–White, cardiac conduction block, diabetes mellitus, peripheral neuropathy, gait disturbance depression, bipolar disorder, ragged red fibres on muscle biopsy	Ptosis, cataracts, optic atrophy, optic neuropathy, progressive external ophthalmoplegia, inherited retinal degeneration
Maternally-inherited Diabetes and Deafness (MIDD) syndrome (520000)	*MT-TL1*, *MT-TE*, *MT-TK* and other genes	FSGS; kidney failure	Sensorineural hearing loss and diabetes in adulthood; seizures dysarthria	Inherited retinal degeneration, macular dystrophy, external ophthalmoplegia
Kearns–Sayre syndrome (530000)	Mutations in various mitochondrial genes	FSGS; Fanconi syndrome, renal tubular acidosis, kidney failure	Sensorineural hearing loss, cardiomyopathy, heart conduction defects	Inherited retinal degeneration, ophthalmoplegia, ptosis

References for the ocular features are provided in the Supplementary file

**Table 3:** Common ocular manifestations in genetic forms of FSGS

Ophthalmic feature	Detail	Genes	Disease name
Hypermetropia		*NPHS1*	Nephrotic syndrome type 1
Myopia		*COL4A3-COL4A4*	XL and AR Alport syndrome
		*CRB2*	FSGS-9
		*LAMB2*	Pierson syndrome
		*NPHS1*	Nephrotic syndrome type 1
		*NPHS2*	Nephrotic syndrome type 2
		*WDR73*	Galloway-Mowat syndrome 1
Congenital size anomaly	Buphthalmos	*LAMB2*	Pierson syndrome; nephrotic syndrome type 5
	Microphthalmia	*LAMB2*	Pierson syndrome; nephrotic syndrome type 5
	Megalocornea	*LAMB2*	Pierson syndrome; nephrotic syndrome type 5
	Microcornea	*LAMB2*	Pierson syndrome; nephrotic syndrome type 5
		*LMX1B*	Nail-patella syndrome
Coloboma	Optic disc coloboma	*PAX2*	FSGS-7
Orbit	Hypertelorism	*LMX1B*	Nail-patella syndrome
		*WDR73*	Galloway-Mowat syndrome 1
Lids	Ptosis	*LAMB2*	Pierson syndrome; nephrotic syndrome type 5
		*LMX1B*	Nail-patella syndrome
		*Mito*	MELAS, MIDD and Kearns-Sayre syndrome
		*SGPL1*	Nephrotic syndrome type 14
		*WT1*	*WT1* diseases
	Corkscrew conjunctival arterioles	*GLA*	Fabry disease
Cornea	Recurrent corneal erosions	*COL4A3- COL4A5*	XL and AR Alport syndrome
	Posterior polymorphous corneal dystrophy	*COL4A3- COL4A5*	XL and AR Alport syndrome
	Corneal verticillata	*GLA*	Fabry disease
	Corneal clouding	*LAMB2*	Pierson syndrome; nephrotic syndrome type 5
	Hazy cornea	*WT1*	*WT1* disease
	Posterior embryotoxon	*LAMB2*	Pierson syndrome; nephrotic syndrome type 5
	Sclerocornea	*LMX1B*	Nail-patella syndrome
	Keratoconus	*LMX1B*	Nail-patella syndrome
	Fuchs endothelial corneal dystrophy	*KANK4* (GWAS)	FSGS
Anterior segment malformations	Iris malformations	*LAMB2*	Pierson syndrome; nephrotic syndrome type 5
	Pigment dispersion syndrome	*COL4A3-COL4A5*	XL and AR Alport syndrome
		*LMX1B*	Nail-patella syndrome
	Ectropion uvea	*LAMB2*	Pierson syndrome; nephrotic syndrome type 5
	Lester’s sign	*LMX1B*	Nail-patella syndrome
	Aniridia	*WT1*	*WT1* diseases
Lens	Anterior lenticonus	*COL4A3-COL4A5*	XL and AR Alport syndrome
	Posterior lenticonus	*COL4A3-COL4A5*	XL and AR Alport syndrome
		*LAMB2*	Pierson syndrome; nephrotic syndrome type 5
	Cataract	*COL4A3-COL4A5*	XL and AR Alport syndrome
		*GLA*	Fabry disease
		*LAMB2*	Pierson syndrome; nephrotic syndrome type 5
		*LMX1B*	Nail-patella syndrome
		*Mito*	MELAS syndrome
		*MYH9*	Macrothrombocytopenia and granulocyte inclusions with or without nephritis or sensorineural hearing loss
		*NPHS1*	Nephrotic syndrome type 1
		*NPHS2*	Nephrotic syndrome type 2
		*SGPL1*	Nephrotic syndrome type 14
		*WT1*	*WT1* diseases
	Spherophakia	*COL4A3- COL4A5*	XL and AR Alport syndrome
		*LAMB2*	Pierson syndrome; nephrotic syndrome type 5
	Microphakia	*LAMB2*	Pierson syndrome; nephrotic syndrome type 5
		*LMX1B*	Nail-patella syndrome
Optic disc	Glaucoma	*LMX1B*	Nail-patella syndrome
		*PAX2*	FSGS-7
		*PLCE1*	Nephrotic syndrome type 3
		*WT1*	*WT1* diseases
	Optic atrophy	*COQ2*	Coenzyme Q10 deficiency, primary, 1
		*COQ6*	Coenzyme Q10 deficiency, primary, 6
		*CRB2*	FSGS-9
		*Mito*	MELAS syndrome
		*NUP93*	Nephrotic syndrome type 12
		*PAX2*	FSGS-7
		*WDR73*	Galloway-Mowat syndrome 1
		*WT1*	*WT1* diseases
	Hypoplastic optic nerve	*LAMB2*	Pierson syndrome; nephrotic syndrome type 5
	Optic neuropathy	*Mito*	MELAS syndrome
		*WDR73*	Galloway-Mowat syndrome 1
	Optic disc pit	*PAX2*	FSGS-7
Macula	Macular hole	*COL4A3- COL4A5*	XL and AR Alport syndrome
	Macular pit	*CRB2*	FSGS-9
	Macular choroidal neovascularisation	*GLA*	Fabry disease
	Macular hypoplasia	*LAMB2*	Pierson syndrome; nephrotic syndrome type 5
	Vitelliform maculopathy	*COL4A3- COL4A5*	XL and AR Alport syndrome
		*Mito*	MELAS syndrome
Retina	Central and peripheral retinopathy	*COL4A3- COL4A5*	XL and AR Alport syndrome
	Retinitis pigmentation/Inherited Retinal Degeneration	*COQ2*	Coenzyme Q10 deficiency, primary, 1
		*COQ8B*	Nephrotic syndrome type 9
		*LAMB2*	Pierson syndrome; nephrotic syndrome type 5
		*Mito*	MELAS, MIDD, Kearns Sayre syndrome
	Pale retina	*CRB2*	FSGS-9
	Microaneurysms	*G6PC*	Glycogen storage disease Ia
		*GLA*	Fabry disease
	Tortuous retinal vessels	*ACTN4*	FSGS-1
		*GLA*	Fabry disease
		*LAMB2*	Pierson syndrome; nephrotic syndrome type 5
	Central retinal artery occlusion	*GLA*	Fabry disease
	Central retinal vein occlusion	*GLA*	Fabry disease
	Retinal detachment	*LAMB2*	Pierson syndrome; nephrotic syndrome type 5
	Retinal haemorrhage	*G6PC*	Glycogen storage disease Ia
		*MYH9*	Macrothrombocytopenia and granulocyte inclusions with or without nephritis or sensorineural hearing loss
Neuro-ophthalmic	Strabismus	*COQ6*	Coenzyme Q10 deficiency, primary, 6
		*LAMB2*	Pierson syndrome; nephrotic syndrome type 5
		*LMX1B*	Nail-patella syndrome
		*NPHS1*	Nephrotic syndrome type 1
		*NPHS2*	Nephrotic syndrome type 2
		*NUP133*	Galloway-Mowat syndrome 8
		*SGPL1*	Nephrotic syndrome type 14
		*WDR73*	Galloway-Mowat syndrome 1
	Pupil disorders	*LAMB2*	Pierson syndrome; nephrotic syndrome type 5
		*LMX1B*	Nail-patella syndrome
	Nystagmus	*COQ6*	Coenzyme Q10 deficiency, primary, 6
		*LAMB2*	Pierson syndrome; nephrotic syndrome type 5
		*WDR73*	Galloway-Mowat syndrome 1
	Progressive external ophthalmoplegia	*Mito*	MELAS syndrome, MIDD, Kearns-Sayre syndrome

Most of the genes commonly associated with congenital nephrotic syndrome have reported ocular manifestations (*NPHS1*, *NPHS2*, *WT1*, *LAMB2*, *PAX2*, *PLCE1*). Many genes associated with childhood–adolescent FSGS also have ocular abnormalities (*NPHS1*, *NPHS2*, *WT1*, *LAMB2*, *SMARCAL1*, *NUP107*, *PLCE1* but not *TRPC6*). The commonest genes associated with adult-onset FSGS (*COL4A3*, *COL4A4*, *COL4A5*) have ocular abnormalities as does *GLA* (Fabry disease), but not *ACTN4*, *CD2AP*, *INF2* or *TRPC6*. *LMX1B* and *MYH9* are infrequent causes of FSGS but ocular features are common.

Mitochondrial diseases that result in FSGS (MELAS, MIDD, Kearn’s Sayre disease) typically result in inherited retinal degeneration and retinal atrophy (Table 2, Fig. 4).

The Renal proteinuria gene list corresponded to some genetic kidney diseases (CAKUT, tubulopathies, ciliopathies) that result in secondary FSGS such as Dent disease (*CLCN5*, *OCRL*), nephronophthisis (*TTC21B*, *NPHP4*), ADTKD-*UMOD* and Imerslund-Grasbeck syndrome 1 (*CUBN*). Of these, Dent disease and nephronophthisis both have ocular features.

Of the 55 genes studied, 51 (93%) had transcripts expressed in the retina, but only 16 (29%) had more than 10 transcripts per million, expression was not examined in other parts of the eye and protein levels were not quantitated. Twenty-seven of these genes (49%) were associated with an ocular phenotype in a mouse model. This meant that a further 12 genes (22%) had > 10 transcripts per million in the retina or an ocular phenotype in a mouse model, suggesting that an uncommon or milder ocular phenotype, even if not recognised to date, might still be found in human disease.

## Common ocular abnormalities in genetic forms of FSGS

The ocular manifestations associated with the largest number of FSGS-causing genes in this search were ptosis, myopia, strabismus, cataract, retinal atrophy and inherited retinal degeneration (Table 3).

Some ocular features were found only associated with one gene and were common and highly specific . These included Pierson syndrome (*LAMB2* variants resulting in microphthalmia); MELAS and MIDD ( mitochondrial variants with inherited retinal degeneration); papillorenal syndrome (*PAX2*, with abnormal disc vasculature); and the Wilm’s tumour, Aniridia, genitourinary anomalies and impaired intellectual development (WAGR) syndrome** (**, *WT1*, with aniridia); Alport syndrome (*COL4A3*, *COL4A4*, *COL4A5*, with lenticonus and fleck retinopathies, temporal retinal thinning, maculopathy and macular hole); and Fabry disease (*GLA*, with corneal verticillata, and tortuous vessels) (Figs. 2, 3 and 4).

Inherited retinal degeneration and retinal atrophy occurred with pathogenic variants in *LAMB2* and with mitochondrial variants (*COQ2*, *COQ8B*, MELAS, MIDD, Kearns–Sayre disease) (Fig. 4). Inherited retinal degeneration is a heterogenous group of diseases characterised by premature loss of photoreceptors (rods, cones, or both ) or the underlying choroid. Clinical features include night blindness, loss of peripheral vision and subsequent loss of central vision. In general, there is no effective treatment but gene therapy has been recently approved for some of these diseases [25].

### Pierson syndrome (*LAMB2*)

*LAMB2* mutations cause both the more severe disease, Pierson syndrome, as well as nephrotic syndrome type 5. Pierson syndrome presents with congenital nephrotic syndrome that progresses to kidney failure. It is associated with neurodevelopmental delay, and kidney biopsies typically demonstrate diffuse mesangial sclerosis. Individuals with FSGS due to *LAMB2* missense variants have a milder phenotype with a slower progression to kidney failure, no neurodevelopmental abnormalities and fewer and milder ocular features [26, 27].

Anophthalmia and microphthalmia are characteristic of Pierson syndrome [27, 28]. Microcoria may be present from birth and characterised by fixed pupils unresponsive to mydriatics [29]. This is likely attributable to iris stroma hypoplasia which appears as a flat, featureless, transilluminable iris, as well as uveal ectropion [27, 29]. Management may require pupilloplasty to improve vision [30].

Other abnormalities also affect vision. Shallow anterior chambers predispose to glaucoma from angle closure or anterior segment dysgenesis [29]. Retinal detachment, scarring and subretinal fibrosis may result in visual loss [31–33].

Nephrotic syndrome type 5 or FSGS resulting from milder *LAMB2* variants and presenting in later childhood or adolescence is associated with more subtle ocular abnormalities often with no visual consequences.

### Papillorenal syndrome (*PAX2*)

Pathogenic variants in *PAX2* commonly result in the papillorenal syndrome which more typically manifests as CAKUT with ocular anomalies. However, certain *PAX2* variants are also associated with SRNS, and adult-onset FSGS rather than CAKUT.

Affected individuals may have unilateral or bilateral optic disc anomalies that vary from an optic disc pit to a chorioretinal coloboma [34–38]. The characteristic feature is the emergence of the retinal vessels from the periphery rather than the centre of the optic disc. Reduced visual acuity correlates with the degree of foveal involvement. The anomaly may vary in each eye and in different affected family members. The visual consequences also vary. Refractive error is common [39]. Complications include perifoveal splitting, optic nerve atrophy and bilateral glaucomatous cupping [34, 35]. There is no treatment.

#### WAGR (*WT1*)

*WT1* mutations result in FSGS (nephrotic syndrome type 4), as well as the WAGR, Denys–Drash and Frasier syndromes. These are very rare diseases that present with proteinuria and nephrotic syndrome in the first years of life, as well as extra-renal features.

Aniridia also occurs in the WAGR syndrome that is associated with hemizygosity for the *PAX6* gene and deletions of 11p13 including *WT1* [40]. Affected individuals have photophobia and reduced visual acuity [41]. Diagnosis of the syndrome is important because of the risk of Wilm’s tumour.

### Alport syndrome (*COL4A3, COL4A4, COL4A5*)

Pathogenic variants in *COL4A3*, *COL4A5* and *COL4A5* result in XL (*COL4A5*) and AR Alport syndrome (where there are two *COL4A3 or COL4A4* variants) which are characterised by kidney failure, sensorineural hearing loss and lenticonus, recurrent corneal erosions and a fleck retinopathy, retinal thinning and macular holes [42]. Proteinuria and FSGS are present in the most boys with XL Alport syndrome and boys and girls with AR disease [43]. AD Alport syndrome with heterozygous *COL4A3* or *COL4A4* variants typically results in haematuria, and sometimes FSGS but without a hearing loss or ocular abnormalities [43, 44].

Corneal erosions occur unilaterally or bilaterally, causing ocular pain or irritation, lacrimation and photophobia [45]. Posterior polymorphous dystrophy is very rare and demonstrated with slit lamp examination [46]. Lesions typically occur on the posterior corneal surface as clear vesicles surrounded by a thickened Descemet’s membrane [46, 47].

Anterior lenticonus is pathognomonic for Alport syndrome and identified on slit lamp examination since the anterior axial projection of the central lens produces an ‘oil droplet’ appearance of the red reflex [48, 49]. Sometimes, a cataract forms after rupture of the lens capsule [50]. Affected individuals have difficulty focusing and reduced visual acuity [51]. Treatment is lens extraction and replacement [52]. Lenticonus does not recur and posterior lenticonus is less common [53].

A fleck retinopathy is the commonest ocular finding in males and females with XL or AR Alport syndrome [54]. The central retinopathy is characterised by multiple white flecks that spare the fovea and is evident on ophthalmoscopy and retinal imaging [55]. The macular reflex may be absent [49]. A peripheral retinopathy appears as larger coalescing lesions that spare the retinal vessels [49]. Visual acuity is not affected, and no treatment is required [21].

Temporal retinal thinning (> 10% of average nasal thickness) is typical on optical coherence tomography in males with XL and in males and females with AR Alport syndrome. Sometimes multiple lamellar holes coalesce to form a ‘giant’ macular hole with loss of central vision [56, 57]. Surgical repair is generally difficult [58] and the patient is typically left with a permanent visual loss [21].

Boys with XL Alport syndrome may have no ocular manifestations, but those who develop kidney failure at a young age will often have a central and peripheral retinopathy, and both lenticonus and temporal retinal thinning have been reported in childhood. Girls with XL Alport syndrome usually have no ocular manifestations. Boys and girls with AR Alport syndrome may have the retinopathy and temporal retinal thinning.

### Fabry disease (*GLA*)

Fabry disease is an XL disorder caused by pathogenic variants in *GLA* which encodes α-galactosidase. The subsequent accumulation of glycosphingolipids leads to end-organ damage. FSGS manifests as proteinuria but kidney failure usually occurs in adulthood. Extrarenal features include acroparaesthesiae, hypohydrosis and abdominal pain from childhood, angiokeratomas, cardiac hypertrophy and cerebrovascular disease. Uncommonly, FSGS occurs without extrarenal features.

Ocular abnormalities are more common in hemizygous males than heterozygous females [59, 60]. Lacrimal secretions are sometimes reduced [61] and potentially contribute to complaints of sore dry eyes [62]. Bilateral conjunctival and retinal vessel tortuosities occur in nearly all affected males and many females [60]. Corkscrew arterioles with irregular dilatations, constrictions and microaneurysms are seen, especially in the inferior conjunctivae. The presence of conjunctival tortuosity correlates with increased disease severity, as measured by the Fabry Outcome Survey-Mainz Severity Score Index (FOS-MSSI), and affected individuals have a more rapid decline in kidney function and increase in cardiac size with age [63]. Tortuous vessels also occur on the upper eyelids [64]. Enzyme replacement therapy may prevent progression of conjunctival and upper lid tortuosity [65] and slow the development of kidney and cardiac disease.

Corneal verticillata are subtle, fine, subepithelial streaks running from the centre of the cornea in whorls to the periphery [66–68]. They develop early in life, are usually located inferiorly and vary from creamy white to golden brown [68]. A brownish, grey or white subepithelial corneal haze may be seen [61]. These corneal changes progress and become more evident with time [69]. Visual acuity is preserved but there may be reduced night vision and increased glare [62, 70]. These features are highly specific for Fabry disease after pharmacological causes such as amiodarone, chloroquine [71], and chlorpromazine use [68] have been excluded [72]. The verticillata do not correlate with disease progression [63] and may regress with enzyme replacement therapy [73, 74].

A granular anterior capsular lens opacity frequently occurs bilaterally in the inferior quadrants [68, 75]. These are usually wedge-shaped, with bases towards the equator and apices towards the centre of the anterior capsule [68]. Posterior lens anomalies are less common, but a cataract with thin, branching, spoke-like opacities radiating from the centre of the posterior capsule is pathognomonic for Fabry disease [67] and may be the only ocular manifestation [68]. Cataracts continue to develop despite enzyme replacement therapy [65].

Retinal vascular changes are also more frequent in hemizygotes [68]. Arterioles appear narrowed with arteriovenous nicking at the peripheries; and capillary micro- and macro-aneurysms occur throughout the retina [75]. Corkscrew tortuosity, especially of the veins, is also seen at the posterior pole [67]. These changes continue to worsen despite enzyme treatment [65].

Further serious ocular complications include central retinal artery occlusion [68, 76, 77], anterior ischemic optic neuropathy [78, 79] and central retinal vein occlusion [80].

### Nail-patella syndrome (*LMX1B*)

Pathogenic variants in *LMX1B* result in the Nail-patella syndrome which is associated with haematuria, and sometimes kidney failure as well as cataract, hearing loss, limb and pelvic skeletal abnormalities [81, 82]. Interestingly, however, many individuals with Nail-patella syndrome have only kidney manifestations [83–86], and, in particular, no ocular features [87].

## Ocular abnormalities in mitochondrial causes of FSGS

### MELAS and MIDD

These diseases result from various pathogenic variants in mitochondrial DNA and are not detected with whole exome sequencing (Table 3).

The m.3242A > G variant is responsible for many individuals with MELAS [88]. The characteristic features have an onset prior to age 40, typically with stroke, seizures, lactic acidosis and ragged red fibres on muscle biopsy [89]. FSGS presents in adulthood and varies from non-nephrotic range proteinuria [17, 90] to SRNS progressing to kidney failure requiring haemodialysis or transplantation [91, 92]. The most common ocular manifestation is a progressive, bilateral macular dystrophy at the level of the retinal pigment epithelium [93, 94]. Lesions first become obvious in adulthood [91, 95] and are graded on ophthalmoscopy and fundus autofluorescence findings. On fundoscopy, mild pigmentary abnormalities initially occur in the central fundus. With disease progression, isolated or multifocal whitish-yellow or hyperpigmented subretinal deposits are seen at the posterior pole [95]. Discontinuous areas of pronounced chorioretinal atrophy develop circumferentially around the fovea and coalesce over time [95–97]. Finally, the central fovea is also affected by extensive chorioretinal atrophy [95]. Visual features include blind spots, impaired night vision and photophobia [94]. Vision deteriorates over time but is relatively preserved if the fovea is spared [95]. Proteinuria precedes the macular dystrophy by years [91].

The bilateral vitelliform macular lesions may develop areas of retinal pigment atrophy over a decade [95, 98]. There is no specific treatment for mitochondrial disease but exercise, reduced alcohol intake, smoking cessation and vitamin supplementation are often used although without much evidence for efficacy [99, 100].

MIDD is a mitochondrial disorder characterised by hearing loss and type 2 diabetes in adulthood. There may be additional features such as cardiomyopathy, myopathy, FSGS or other kidney disorders and neuropsychiatric features [101]. MIDD accounts for up to 1% of all new cases of diabetes [102]. It results from a pathogenic variant that impairs oxidative phosphorylation and ATP production.

The ocular features include ptosis and inherited retinal degeneration which occur in almost all the patients [103].

### Kearns–Sayre syndrome

This is another mitochondrial disease with variable features including FSGS or renal tubulopathy, short stature, microcephaly, myopathy, cardiomyopathy, cardiac conduction defect, hearing loss and cerebellar ataxia. The typical ocular abnormalities include progressive external ophthalmoplegia, ptosis and inherited retinal degeneration.

## Discussion

Thirty-two of the 55 genes from the Genomics England Renal proteinuria panel were associated with an ocular phenotype in human disease. A further 12 genes were expressed in the retina or the corresponding mouse models had ocular features, suggesting that additional ocular manifestations might still be identified. Thus, at least 44 of the 55 genes (80%) currently recognised to be affected in genetic forms of FSGS may have ocular abnormalities.

The commonest reported ocular associations of genetic FSGS genes are cataracts, myopia, strabismus, ptosis, retinal atrophy and inherited retinal degeneration, but these features are often found infrequently. In contrast, some ocular abnormalities are associated with only one gene, but the diseases themselves are relatively common causes of genetic FSGS. These abnormalities include the optic disc anomalies with papillorenal syndrome, the fleck retinopathy in Alport syndrome and the corneal verticillata and vascular tortuosity with Fabry disease. In the mitochondrial diseases, inherited retinal dystrophy and retinal atrophy are often present but the kidney manifestations including FSGS, tubulopathies and cysts are more variable.

In general, it is not possible to deduce how often ocular abnormalities occur in individual cases of genetic forms of FSGS. The likelihood of ocular abnormalities depends on the age of the individual, the gene affected and the variant type, that is, whether it is a nonsense or missense change. The ocular features a may be different in other affected family members. In addition, the demonstration of an ocular abnormality will depend on a thorough ophthalmic examination. Mild or early manifestations may be obvious only with a formal ophthalmic review or investigations such as optical coherence tomography (OCT) or peripheral retinal imaging.

Structural ocular abnormalities such as coloboma, optic disc anomaly or microphthalmia are typically present from birth, and treatment is not usually possible. Strabismus becomes obvious early. The ocular features of Fabry disease may not be present at the time of kidney disease diagnosis, but develop over time, and treatment slows progression [65, 69]. With pathogenic variants in the mitochondrial genome, the kidney disease often precedes the onset of atrophy or inherited retinal degeneration [91].

Many of the genetic kidney diseases previously considered to be paediatric are now also diagnosed for the first time in adults but the ocular manifestations may be less pronounced than those seen in disease with a childhood onset. Thus, Pierson syndrome may manifest with anophthalmia or microphthalmia in neonates but with renal-limited FSGS in adolescents or adults. Nevertheless, it is important for paediatric nephrologists to understand how ocular manifestations in genetic kidney disease differ with increasing age because they may be able to make the diagnosis in an older family member.

The presence of ocular abnormalities may also indicate more severe kidney disease. In Pierson syndrome, severe ocular phenotypes are associated with earlier onset kidney failure [26–28]. In Alport syndrome, the more severe genetic variants are associated more often with lenticonus, more severe central retinopathy and temporal atrophy, and earlier onset kidney failure [21, 104, 105]. In Fabry disease, the presence of retinal and conjunctival vessel tortuosity correlates with a more rapid decline in kidney and cardiac function [63].

Ocular phenotypes are still not described for some of the FSGS-associated genes, even where there is retinal expression or a mouse ocular phenotype. However, many of these diseases are very rare, patients may not have undergone formal ophthalmological review; the reporting laboratory may not have had access to the clinical examination findings; the association with the ocular abnormality may not have been recognised; and sometimes, conversely, the reported ocular features are coincidental.

This study has tested for ocular abnormalities as indicators of extrarenal disease that suggest a genetic basis for FSGS, but other organ systems, such as the hearing, heart, skeleton and brain, are commonly affected too. These abnormalities may be more obvious than the ocular features. Nevertheless, a basic ophthalmic examination is inexpensive and non-invasive, and some ocular abnormalities affect vision and must be treated or monitored for complications.

Some ocular features such as coloboma and optic atrophy are obvious to the renal physician and their association with FSGS suggests a genetic cause. In such cases, it is worthwhile seeking a family history of kidney disease and undertaking genetic testing. An early assessment by an interested ophthalmologist where ocular involvement is suspected is important. Even where the abnormality is present at birth and does not typically progress, complications such as strabismus, cataract, glaucoma and retinal detachment can occur. The ophthalmologist is best placed to assess how often the ophthalmic features should be monitored and any treatment required, such as surgery for strabismus or retinal detachment. The patient must be assessed for other syndromic features and first degree relatives also examined.

The strengths of this study were the examination of the Genomics England Renal proteinuria panel which is widely used in testing for the genetic cause of FSGS, and the systematic approach to identifying ocular abnormalities from OMIM and the literature as well as examining retinal expression and the effect in mouse models. Genes associated with FSGS will continue to be identified, and some of these will have ocular features. However, the aim of this study was not to identify all the FSGS genes with an ocular association, but rather to determine whether ocular features were common enough to identify a genetic cause, and the gene itself, whether they indicated more severe disease and whether they affected vision.

The study’s major limitations were that the Renal proteinuria panel did not include some genes that might be considered pathogenic. In addition,some reports of genetic FSGS were rare; the ophthalmic examinations often absent or incomplete; and sometimes a gene was not only associated with FSGS but also with CAKUT, a tubulopathy or cystic kidney disease [8, 106]. Finally, there was little data on how often the ocular manifestations were present in affected children and adults and the age by which features had developed if not apparent at birth. 

In conclusion, ocular abnormalities are common in genetic forms of FSGS, suggest their genetic nature and often a specific diagnosis, and may predict renal disease severity. Importantly, some genetic forms of FSGS are associated with ocular features that must be monitored and treated to avoid complications and to maintain vision.

### Supplementary Information

ESM 1 (PDF 338 kb)

## Data Availability

All data used in this study is available in the manuscript or the Supplementary file.
