# Supplementary material for: Ocular manifestations of the genetic causes of focal and segmental glomerulosclerosis
Source: Pediatr Nephrol. 2023 Aug 14;39(3):655–79. doi: 10.1007/s00467-023-06073-y (PMC10817844; doi:10.1007/s00467-023-06073-y)
Supplement: Supplementary file 1 — (PDF 338 kb) [file 467_2023_6073_MOESM1_ESM.pdf]

Supplementary Tables

Suppl Table 1. Genetic FSGS, ocular associations, retinal expression, and ocular phenotypes in mouse models

Suppl Table 2: Common mitochondrial diseases causing FSGS and their ocular manifestations. This corresponds to Table 2 in the manuscript with references

Suppl Table 1. Genetic FSGS, ocular associations, retinal expression, and ocular phenotypes in mouse models

**Gene (OMIM)**	**Disease (OMIM)**	**Function**	**Kidney features (OMIM)**	**Extrarenal features (OMIM)**	**Ocular features**	**Retinal mRNA expression** Human Protein Atlas [1]	**Mouse Model** [2]
ACTN4 (604638) (GE- green)	FSGS-1 (603278) AD	Cytoskeleton	FSGS	None reported	Retinal venular tortuosity (GWAS) [3]	26.0 TPM	Abnormal eye muscle, lens, optic cup, retina
AMN (605799) (GE- amber)	Imerslund-Grasbeck syndrome 2 (618882) AR	Trans-membrane protein	Mild proteinuria	Malabsorption of vitamin B12, megaloblastic anaemia	Anterior polar cataract, posterior subcapsular cataract [4]	0 TPM	Microphthalmia
ANLN 616027 (GE- amber)	FSGS-6 (616032) AD	Cytoskeleton	FSGS	None reported	None reported	0.3 TPM	No eye pathology noted [2][2][2][2][2][2][2][2][2][2][2][2][2][2][2][2]
ARHGDIA (601925) (GE- green)	Nephrotic syndrome type 8 (6152440 (AR)	Rho-GDP dissociation inhibitor	Proteinuria, FSGS	Hearing loss, intellectual disability	None reported	14.6 TPM	No eye pathology noted
CD151 602243 (GE- amber)	Epidermolysis bullosa simplex 7, with nephropathy and deafness (609057) AR	Transmembrane protein	Kidney agenesis, proteinuria, nephrotic syndrome	Hearing loss, pretibial bullae	Bilateral lacrimal duct stenosis	9.6 TPM	No eye pathology noted
CD2AP 604241 (GE - amber)	FSGS – 3 (607832) AR	Slit diaphragm complex	FSGS	None reported	None reported	6.9 TPM	No eye pathology noted [2][2][2][2][2][2][2][2][2][2][2][2][2][2][2][2]
CLCN5 (300008) (GE- green)	Dent disease 1 (300009); Proteinuria (308990) XL	Proximal renal tubular defect	Decreased phosphate reabsorption; stones, proteinuria	Rickets, osteomalacia	Cataract	3.4 TPM	No eye pathology noted

COL4A3 *(120070)*<br><br>*COL4A4* *(120131)*<br><br>*COL4A5* (303630) (GE- all green)	Alport syndrome XL, AR	Glomerular basement membrane and receptors, cell matrix	Haematuria, proteinuria, FSGS	Sensorineural hearing loss<br><br>*COL4A5* variants only - Leiomyomata in trachea, bronchi, female genitalia; aortic, cerebral and coronary artery aneurysms	Myopia [5], recurrent corneal erosions [6], posterior polymorphous corneal dystrophy [7, 8], corneal arcus [9], pigment dispersion syndrome [10], anterior lenticonus [11], cataract [12], anterior polar cataract [13], posterior lenticonus [14], central retinopathy [15], peripheral retinopathy [5], bull's eye maculopathy [16], macular holes [17, 18], temporal retinal thinning [19], retinal lozenge [20], vitelliform maculopathy [16]	52.9 TPM; 8.4 TPM; 6.1 TPM	Abnormal lens, abnormal eye physiology; irregularly shaped pupil; cataract [2][2][2][2][2][2][2][2][2][2][2][2][2][2][2][2]
COQ2 (609825) (GE- green)	Coenzyme Q10 deficiency, primary, 1 (607426) AR	Mitochondrial function	FSGS, diffuse mesangial sclerosis	Cerebellar ataxia, myoglobinuria, progressive muscle weakness, lactic acidaemia, encephalopathy, hypotonia, psychomotor delay, seizures, hypertrophic cardiomyopathy	Optic atrophy, Inherited retinal degeneration [21]	4.0 TPM	No eye pathology noted [2][2][2][2][2][2][2][2][2][2][2][2][2][2][2][2]
COQ6 (614647) (GE- green)	Coenzyme Q10 deficiency, primary, 6 (614650) AR	Mitochondrial function	FSGS, diffuse mesangial sclerosis	Seizures, ataxia, facial dysmorphism, sensorineural hearing loss	Bilateral optic nerve atrophy, exotropia with nystagmus [22]	5.8 TPM	Abnormal cornea, abnormal vitreous [2][2][2][2][2][2][2][2][2][2][2][2][2][2][2][2]
COQ8B (615567) (GE- green)	Nephrotic syndrome type 9 (615573) (AR)	Mitochondrial function	FSGS, collapsing FSGS	Mild intellectual disability, seizures, thoracic aortic aneurysm	Inherited retinal degeneration [23]	1.3 TPM	No eye pathology noted [2][2][2][2][2][2][2][2][2][2][2][2][2][2][2][2]
CRB2 (609720) (GE- green)	FSGS-9 (616220) AR	Slit diaphragm complex	FSGS, cysts	Ventriculomegaly, aqueductal stenosis, cerebellar hypoplasia, seizures, quadrigeminal cysts, Atrial or ventricular septal defect, scimitar syndrome	Myopia, optic atrophy, pale retina, macular pit [24]	8.1 TPM; high in limiting membrane	Retinal degeneration [2][2][2][2][2][2][2][2][2][2][2][2][2][2][2][2]
CUBN *(602997)* (GE- green)	Imerslund-Grasbeck	Intestinal receptor for Intrinsic factor	Proteinuria	B12 malabsorption, Megaloblastic anaemia	None reported	3.0 TPM	Fused cornea and lens, iris synechiae,

	syndrome 1 (261100), AR						abnormal iris morphology
DGKE 601440 (GE- amber)	Nephrotic syndrome type 7 (615008) AR	Intracellular lipid kinase	Nephrotic syndrome, atypical haemolytic uremic syndrome (in some)	Haemolytic anaemia, thrombocytopenia in some patients	None reported	34.5 TPM	No eye pathology noted
DLC1 (604258) (GE- green)	SSNS and SRNS, AR	Rho GTPase	Proteinuria	None reported	None reported	1.5 TPM	No eye pathology noted
EMP2 (602334) (GE- green)	Nephrotic syndrome type 10 (615861) AR	Tetraspanin protein	Proteinuria	None reported	None reported	2.0 TPM	No eye pathology noted
FAT1 (600976) (GE- green)	Proteinuria	Cadherin-like protein	Glomerulotubular nephropathy	None reported	None reported	3.4 TPM	Aniridia, aphakia, microphthalmia, Inherited retinal degeneration, abnormal retinal vasculature
FN1 135600 (GE- amber)	Glomerulopathy with fibronectin deposits 2 (601894) AD	Glycoprotein on cell surface and in fluids	Proteinuria, kidney failure	None reported	None reported	6.0 TPM	No eye pathology noted
GLA 300644 (GE – amber list)	Fabry disease (301500) XL	Galactosidase	Fabry disease	Angiokeratomas; acroparaesthesia; cardiac hypertrophy; hypohydrosis, mitral valve prolapse; episodic diarrhoea; abdominal pain; cerebrovascular disease; dysmorphic facial and extremity features	Conjunctival lymphangiectasia [25]; reduced lacrimal secretion [26]; upper lid vessel tortuosity [27]; corkscrew arterioles on conjunctivae [28]; corneal verticillata [29]; fine brown, subepithelial corneal lines [26]; anterior capsule lens opacification[28]; wedge-shaped lens opacities [30]; branching, spoke-like posterior subcapsular cataract [31]; optic neuritis [32]; corkscrew tortuosity of retinal vessels [31]; retinal capillary micro and macroaneurysms [28]; retinal oedema [29]; central retinal artery occlusion; anterior ischemic optic neuropathy [33,	6.9 TPM	No eye pathology noted

					34]; central retinal vein occlusion [35]; macular choroidal neovascularisation [36]; blind spot enlargement [26]		
INF2 (610982) *C14ORF173* (GE- green); *INF2* (610982) *C14ORF173* (GE- green)	Charcot-Marie-Tooth disease, dominant intermediate E; FSGS-5 (613237)	Cytoskeleton, mitochondrial function	FSGS; FSGS	'Claw hand' deformities, symmetrical muscle atrophy, peripheral nerve dysfunction, moderate-severe muscle weakness, sensorineural hearing loss	None reported	3.3 TPM	No eye pathology noted [2][2][2][2][2][2][2][2][2][2][2][2][2][2][2][2]
ITGA3 (605025) *GAPB3* (GE- green)	Interstitial lung disease, nephrotic syndrome, and epidermolysis bullosa, congenital; ILNEB (614748) AR	Glomerular basement membrane and receptors	FSGS	Skin fragility; blisters; sparse scalp hair, eyebrows and eyelashes; nail dystrophy; distal onycholysis following mild trauma; respiratory distress; diffuse interstitial lung disease; epidermolysis bullosa; mitral insufficiency	None reported	4.4 TPM	No eye pathology noted [2][2][2][2][2][2][2][2][2][2][2][2][2][2][2][2]
ITSN1 (602442) (GE- green)	Steroid-sensitive nephrotic syndrome, AR		Proteinuria	None reported	None reported	28.5 TPM	No eye pathology noted
KANK2 614610 (GE-amber)	Nephrotic syndrome type 16 (617783) AR	Regulate actin polymerisation	Nephrotic syndrome, steroid sensitive	None or with palmoplantar keratoderma and woolly hair (OMIM 616099)	None reported	19.0 TPM	Eye pathology noted
LAGE3 (300060) (GE- green)	Galloway-Mowat syndrome (301006) XL	Not known	Nephrotic syndrome, SSNS, kidney failure	Microcephaly, intellectual disability, seizures	Nystagmus	4.6 TPM	No eye pathology noted
LAMA5 601033 (GE – amber)	Nephrotic syndrome 26 (620049) AR	Component of glomerular basement membrane	Nephrotic syndrome, kidney failure	Scarring of the skin, muscle weakness, ligamentous laxity, malabsorption and hypothyroidism	Night blindness	0.6 TPM	Microphthalmia, anophthalmia

LAMB2 (150325) (GE- green);<br><br>*LAMB2* (150325) (GE- green)	Nephrotic syndrome type 5 (614199) AR;<br><br>Pierson syndrome (609049) AR	GBM and receptors [37], cell matrix;<br><br>GBM and receptors, cell matrix	FSGS;<br><br>FSGS diffuse mesangial sclerosis, minimal change disease, membranous glomerulonephritis	Delayed motor milestones, severe proximal limb muscle weakness, scoliosis;<br><br>Neurodevelopmental delay, hypotonia, progressive microcephaly, scoliosis, genu varum	Ptosis [38], myopia, hypopigmented retina [39], hypoplastic macular areas, poor foveal reflex, small pupils, limited external ocular movements [38], nystagmus, strabismus [39];<br><br>Microphthalmos; high myopia; posterior staphyloma [40]; microcornea [41]; megalocornea clouded cornea [40]; band keratopathy[42]; anterior segme dysgenesis; shallow anterior chamber; iris hypoplasia; enlarge iris vessels; posterior synechiae; ectropion uvea [40]; remnants of pupillary membranes [43]; spherophakia; microphakia[40]; posterior lenticonus; cataract [44 hypoplastic optic nerve [40]; hypopigmented fundus; macular hypoplasia; retinal detachment; tortuous retinal vessels [45];; Inherited Retinal Degeneration; vitreoretinal adhesions and tractions; atrophic choriocapillari [42]; microcoria [44]; nystagmus [45]; strabismus [43]	11.7 TPM	Abnormal retina [2][2][2][2][2][2][2][2][2][2][2][2][2][2][2][2][2]
LCAT 606967 (GE- amber)	Norum disease (245900) AR	Enzyme	Proteinuria, kidney failure	Haemolytic anaemia	Corneal lipid deposits, corneal opacities [46]	2.8 TPM	No eye pathology noted
LMX1B (602575) (GE- green)	Nail-patella syndrome (161200) AD	DNA repair, transcription, Nuclear transport	Diffuse hyaline thickening of GBM, FSGS, FSGS without extrarenal involvement	Nail anomalies, triangular lunulae, swan neck deformity of fingers, absent creases on dorsal distal interphalangeal joints of fingers, scoliosis, patellofemoral joint anomalies, elbow anomalies, pelvis anomalies, sensorineural hearing loss; weak, crumbling teeth;	Ptosis [47], microcornea, sclerocornea [47], keratoconus [47], corneal oedema [48], microphakia [47], congenital cataract [49], cloverleaf hyperpigmentation around central iris (Lester's sign) [50], anterior synechiae [48], glaucoma, ocular hypertension [50], multiple iris processes [49],	0 TPM	Abnormal iris, abnormal cornea, corneal ulcers, abnormal lens, microphthalmia

				epilepsy, hypertelorism, epicanthal folds	heterochromia [47], pigment dispersion syndrome [51], anisocoria [47], strabismus [52]		
MAGI2 (606382) (GE- green)	Nephrotic syndrome type 15 (617609)	Cell junction organisation	Nephrotic syndrome	None reported	None reported	11.6 TPM	No eye pathology noted
MYH9 (160775) (GE- green)	Macrothrombo -cytopenia, granulocyte inclusions with or without nephritis or sensorineural hearing loss (155100) AD	Cytoskeleton	FSGS [53-55]	Thrombocytopaenia, giant platelets, high frequency sensorineural hearing loss	Congenital cataract [56, 57], presenile cataract [58], neovascularisation, retinal haemorrhage, vitreous haemorrhage, peripheral pigmentary changes [59]	9.7 TPM	Abnormal cornea, abnormal lens [2][2][2][2][2][2][2][2][2][2][2][2][2][2][2][2]
MYO1E (601479) (GE- green)	FSGS-6 (614131) AR	Slit diaphragm complex cytoskeleton	FSGS	None reported	None reported	9.9 TPM	No eye pathology noted
NPHS1 (602716) (GE- green)	Nephrotic syndrome type 1 (256300) AR	Slit diaphragm complex	FSGS, minimal change glomerulonephritis	Pyloric stenosis	Myopia, high hyperopia, bilateral posterior subcapsular cataract, strabismus, amblyopia	0.7 TPM	No eye pathology noted [2][2][2][2][2][2][2][2][2][2][2][2][2][2][2][2]
NPHS2 (604766) (GE- green)	Nephrotic syndrome type 2 (600995) AR	Slit diaphragm complex	FSGS	None reported	Myopic astigmatism, cataract, anisometropic amblyopia, exotropia	0 TPM	No eye pathology noted [2][2][2][2][2][2][2][2][2][2][2][2][2][2][2][2]
NUP85 (170285) *NUP75* (GE- green)	Nephrotic syndrome type 17 (618176) AR	Nuclear proteins and transcription factors	FSGS	Intellectual disability, short stature	None reported	11.2 TPM	No eye pathology noted [2][2][2][2][2][2][2][2][2][2][2][2][2][2][2][2]
NUP93 (614351) (GE- green)	Nephrotic syndrome type 12 (616892) AR	Nuclear proteins and transcription factors	FSGS, diffuse mesangial sclerosis	None reported	None reported	31.7 TPM	No eye pathology noted [2][2][2][2][2][2][2][2][2][2][2][2][2][2][2][2]

NUP107 (607617) (GE- green)	Galloway-Mowat syndrome 7 (618348) AR; Nephrotic syndrome, type 11 (616730) AR	Nuclear proteins and transcription factors	FSGS, IgA nephropathy; FSGS, minimal change nephrotic syndrome, diffuse mesangial sclerosis	Mild-moderate intellectual disability, developmental delay, microcephaly, sloping forehead, bitemporal narrowing, smooth philtrum, micrognathia, simian crease, clinodactyly, right brachial plexopathy, bifid thumb, cubitus valgus, hallux valgus, pectus excavatum, kyphoscoliosis, short stature; Cleft lip, cleft palate	Hypertelorism (OMIM)	14.7 TPM	No eye pathology noted
NUP133 (607613) (GE- green)	Galloway-Mowat syndrome 8 (618349) AR	Nuclear proteins and transcription factors	FSGS	Psychomotor retardation, hypotonia, microcephaly, bilateral thumb deviation, enamel hypoplasia, atopic dermatitis, epilepsy, talipes	Convergent strabismus [60]	19.1 TPM	No eye pathology noted [2][2][2][2][2][2][2][2][2][2][2][2][2][2][2][2]
OCRL (300535) (GE- green)	Dent disease 2; Lowe syndrome, XL	Membrane trafficking	Proximal tubular defect; aminoacid-uria;nephrocalcinosis kidney failure	Developmental delay, intellectual impairment	Cataracts present at birth, glaucoma, microphthalmia, impaired visual acuity, (Lowe syndrome)	9.9 TPM	Abnormal eye
OSGEP (610107) (GE- green)	Galloway-Mowat syndrome 3 (617729) AR		Nephrotic syndrome, kidney failure	Microcephaly, micrognathia, arachnodactyly, intellectual disability	Microphthalmia, strabismus, hypertelorism, impaired vision	4.1 TPM	No eye pathology noted
PAX2 (167409) (GE- green)	FSGS-7 (616002) AD	DNA repair, transcription, nuclear transport	Adult onset FSGS, no CAKUT	Mild hypertrophic cardiomyopathy, microcephaly cryptorchidism	Optic nerve coloboma; optic disc coloboma, optic disc pit, optic nerve atrophy, glaucomatous cupping	1.2 TPM;	Coloboma, abnormal retina, abnormal blood vessels [2][2][2][2][2][2][2][2][2][2][2][2][2][2][2][2]
PDSS2 (610564) (GE- green)	Coenzyme Q10 Deficiency, Primary, 3 (614652) AR	Mitochondrial function	Susceptibility to FSGS	Hypotonia, seizures	Cortical blindness, Leigh syndrome	6.0 TPM	No eye pathology noted
PLCE1 (608414) (GE- green)	Nephrotic syndrome type 3 (610725) AR	Cell signalling, slit diaphragm	FSGS, diffuse mesangial sclerosis	None reported	Glaucoma (GWAS) [61]	7.3 TPM	No eye pathology noted

PODXL (602632) (GE- green)	AR	Cell membrane associated protein	FSGS	None reported	None reported	67.8 TPM	Abnormal retinal pathology
PTPRO 600579 (GE-amber)	Nephrotic syndrome type 6 (614196) AR	Podocyte tyrosine phosphatase	FSGS	None reported	None reported	7.5 TPM	No eye pathology noted
SCARB2 (602257) (GE- green)	Epilepsy, progressive myoclonic 4, with or without kidney failure (254900) AR	Lysosome [62]	FSGS	Progressive fine tremor, action myoclonus, convulsive seizures, ataxia, dysarthria, slight cognitive impairment, slowed horizontal saccades[63], rapid eye movement sleep behaviour disorder (GWAS)	Slowed horizontal saccades	53.2 TPM	No eye pathology noted [2][2][2][2][2][2][2][2][2][2][2][2][2][2][2][2]
SGPL1 (603729) (GE- green)	Nephrotic syndrome type 14 (617575) AR	Metabolic and cytosolic	FSGS, diffuse mesangial sclerosis	Primary adrenal insufficiency, hyperpigmentation, ichthyosis, primary hypothyroidism, neurodevelopmental delay, ataxia, cognitive decline, sensorineural deafness, seizures, lymphopaenia, hypocalcaemia, hypoglycaemia, dyslipidaemia, cryptorchidism, micropenis, microcephaly, hypotonia, peripheral neuropathy	Bilateral cataracts, salt and pepper retinopathy, ptosis, esotropia, amblyopia, strabismus	10.8 TPM	No eye pathology noted [2][2][2][2][2][2][2][2][2][2][2][2][2][2][2][2]
SMARCAL1 (606622) (GE- green)	Schimke immunoosseous dysplasia (242900) AR	DNA repair, transcription, nuclear transport	FSGS	Lymphopenia. multiple lentigines, spondyloepiphyseal dysplasia, cerebral infarcts	Astigmatism, corneal opacity, myopia	5.8 TPM	No eye pathology noted
TBC1D8B (301027) (GE- green)	Nephrotic syndrome type 20 (301028) XL	Metabolic and cytosolic	FSGS, kidney failure	None reported	None reported	9.6 TPM	No eye pathology noted
TPRKB (608680) (GE – amber)	Galloway-Mowat syndrome 5 (617731) AR	Part of KEOPS complex	FSGS, kidney failure	Microcephaly, delayed psychomotor development, demyelination	Hypertelorism, epicanthal folds	16.1 TPM	No eye pathology noted

TNS2 (607717) (GE- green)	Nephrotic syndrome type 22	Binds to actin	Minimal change diffuse mesangial sclerosis	None reported	None reported	6.0 TPM	No eye pathology noted
TP53RK (608679) (GE- green)	Galloway-Mowat syndrome 4 (617730) AR	DNA repair, transcription	FSGS, diffuse mesangial sclerosis	Primary microcephaly, seizures, developmental delay, cognitive impairment, hypotonia, spasticity, facial dysmorphism, short stature, tapered fingers, feeding difficulties, multiple hypo- and hyperpigmented macules	Hypertelorism, visual impairment	6.4 TPM	No eye pathology noted
TRPC6 (603652) (GE- green)	FSGS-2 (603965) AD	Cell signalling, slit diaphragm	FSGS [64-68], minimal change disease, kidney failure	None reported	None reported	0.3 TPM	Abnormal eye physiology
WDR73 (616144) (GE- green)	Galloway-Mowat syndrome 1 (251300) AR	DNA repair, transcription, nuclear transport	FSGS, diffuse mesangial sclerosis [69-71]	Microcephaly, hiatus hernia, hypotonia, abnormal skull, flat occiput, seizures, developmental delay, bilateral club feet, micrognathia, close-set eyes, large ears, small midface, aqueductal stenosis, epicanthal folds, broad nasal bridge, hypertelorism, congenital hypothyroidism, contractures, bilateral simian crease, thyroid dysplasia, adrenal hypoplasia hypertonia, spastic quadriplegia, short stature, dystonia, ataxia, hirsutism, hip dysplasia	Myopia [72], optic atrophy [72, 73], optic neuropathy [74] [75]retinopathy [72], abnormal visual evoked potentials [76], strabismus [72, 76, 77], nystagmus [76], oculomotor apraxia [72]	3.4 TPM	No eye pathology noted
WT1 (607) (GE- green)	*WT1* diseases, FSGS-4 (256370) AD	DNA repair, transcription, nuclear transport	Wilms tumour, FSGS-4 diffuse mesangial sclerosis	Congenital hemihypertrophy, genitourinary abnormalities, mental retardation, diaphragmatic hernia, hypomyelinating leukodystrophy	Bilateral ptosis [78, 79], hazy cornea [80], nuclear and foetal cataracts [78], anterior polar cataracts [80], posterior capsular cataracts [79] increased intraocular pressure [80], aniridia [81-84], bilateral optic atrophy [85]	0 TPM	No eye pathology noted [2][2][2][2][2][2][2][2][2][2][2][2][2][2][2][2]

OMIM – Online Mendelian disease in Man database with genes and corresponding diseases including detailed clinical phenotypes; AD, autosomal dominant; AR, autosomal recessive; CAKUT, congenital anomalies of the kidney and urinary tract; FSGS, focal segmental glomerulosclerosis; GE – Genomics England panel gene; GWAS, genome side association studies; IRD, inherited retinal degeneration; XL, X linked. Genes are indicated from the 'green' or 'amber' lists of the Renal proteinuria panel; TPM transcripts per million

Table 2: Common mitochondrial diseases causing FSGS and their ocular manifestations

Disease (OMIM)	Genes	Kidney features (OMIM)	Extrarenal features (OMIM)	Ocular features
Myopathy, Encephalopathy, Lactic Acidosis, Stroke (MELAS) syndrome	*MT-TL1* and others [86]	FSGS [87-91], tubulointerstitial nephropathy [90], kidney failure	Stroke, seizures, lactic acidosis, dementia, short stature, hearing loss, learning disability, myoclonus, cerebellar signs, congestive heart failure, Wolff-Parkinson-White, cardiac conduction block, diabetes mellitus [92], [93], peripheral neuropathy [94], gait disturbance [95], depression, bipolar disorder, [96], ragged red fibres on muscle biopsy[92]	Ptosis, cataracts [97], optic atrophy [92], optic neuropathy [98], progressive external ophthalmoplegia [99-101], Inherited retinal degeneration [102-108]
Maternally-inherited Diabetes and Deafness (MIDD) syndrome (520000)	*MT-TL1, MT-TE, MT-TK* and others	FSGS; kidney failure [109]	Sensorineural hearing loss and diabetes in adulthood [110]; seizures dysarthria	Inherited retinal degeneration, macular dystrophy, external ophthalmoplegia
Kearns Sayre syndrome (53000)	Mutations in various mitochondrial genes	FSGS; Fanconi syndrome, renal tubular acidosis, kidney failure [111]	Sensorineural hearing loss, cardiomyopathy, heart conduction defects	Inherited retinal degeneration, ophthalmoplegia, ptosis

References

1. Uhlén M, Fagerberg L, Hallström BM, Lindskog C, Oksvold P, Mardinoglu A, Sivertsson Å, Kampf C, Sjöstedt E, Asplund A, Olsson I, Edlund K, Lundberg E, Navani S, Szigyarto CA-K, Odeberg J, Djureinovic D, Takanen JO, Hober S, Alm T, Edqvist P-H, Berling H, Tegel H, Mulder J, Rockberg J, Nilsson P, Schwenk JM, Hamsten M, von Feilitzen K, Forsberg M, Persson L, Johansson F, Zwahlen M, von Heijne G, Nielsen J, Pontén F (2015) Tissue-based map of the human proteome. Science 347:1260419.
2. Bult CJ, Blake JA, Smith CL, Kadin JA, Richardson JE (2019) Mouse Genome Database (MGD) 2019. Nucleic Acids Res 47:D801-d806.
3. Veluchamy A, Ballerini L, Vitart V, Schraut KE, Kirin M, Campbell H, Joshi PK, Relan D, Harris S, Brown E, Vaidya SS, Dhillon B, Zhou K, Pearson ER, Hayward C, Polasek O, Deary IJ, MacGillivray T, Wilson JF, Trucco E, Palmer CNA, Doney ASF (2019) Novel Genetic Locus Influencing Retinal Venular Tortuosity Is Also Associated With Risk of Coronary Artery Disease. Arteriosclerosis, Thrombosis & Vascular Biology 39:2542-2552.
4. Kamath SJ, Amruthavalli KS, Bhat KG, P. K (2017) Imerslund-Grasbeck syndrome with developmental cataract. JCRS Online Case Reports 5:67-68.
5. Govan JA (1983) Ocular manifestations of Alport's syndrome: a hereditary disorder of basement membranes? The British journal of ophthalmology 67:493-503.
6. Burke JP, Talbot JF, Clearkin LG (1991) Recurrent corneal epithelial erosions in Alport's syndrome. Acta Ophthalmologica 69:555-557.
7. Sabates R, Krachmer JH, Weingeist TA (1983) Ocular Findings in Alport's Syndrome. Ophthalmologica 186:204-210.
8. Teekhasaenee C, Nimmanit S, Wutthiphan S, Vareesangthip K, Laohapand T, Malasitr P, Ritch R (1991) Posterior polymorphous dystrophy and Alport syndrome. Ophthalmology 98:1207-1215.
9. Arnott EJ, Crawfurd MD, Toghill PJ (1966) Anterior lenticonus and Alport's syndrome. The British journal of ophthalmology 50:390-403.
10. Davies PD (1970) Pigment dispersion in a case of Alport's syndrome. The British journal of ophthalmology 54:557-561.
11. METTIER SR, JR. (1961) Ocular Defects Associated with Familial Renal Disease and Deafness: Case Reports and Review of Literature. Archives of Ophthalmology 65:386-391.
12. Goldbloom RB, Fraser FC, Waugh D, Aronovitch M, Wiglesworth FW (1957) HEREDITARY RENAL DISEASE ASSOCIATED WITH NERVE DEAFNESS AND OCULAR LESIONS. Pediatrics 20:241-252.
13. Streeten BW, Robinson MR, Wallace R, Jones DB (1987) Lens Capsule Abnormalities in Alport's Syndrome. Archives of Ophthalmology 105:1693-1697.
14. NIELSEN CE (1978) LENTICONUS ANTERIOR AND ALPORT'S SYNDROME. Acta Ophthalmologica 56:518-530.
15. Polak BC, Hogewind BL (1977) Macular lesions in Alport's disease. Am J Ophthalmol 84:532-535.
16. Fawzi AA, Lee NG, Eliott D, Song J, Stewart JM (2009) Retinal findings in patients with Alport Syndrome: Expanding the clinical spectrum. British Journal of Ophthalmology 93:1606-1611.
17. Mete UO, Karaaslan C, Ozbilgin MK, Polat S, Tap O, Kaya M (1996) Alport's syndrome with bilateral macular hole. Acta Ophthalmol Scand 74:77-80.
18. Rahman W, Banerjee S (2007) Giant macular hole in Alport syndrome. Can J Ophthalmol 42:314-315.
19. Usui T, Ichibe M, Hasegawa S, Miki A, Baba E, Tanimoto N, Abe H (2004) Symmetrical reduced retinal thickness in a patient with Alport syndrome. Retina 24:977-979.
20. Colville D, Wang YY, Tan R, Savige J (2009) The retinal "lozenge" or "dull macular reflex" in Alport syndrome may be associated with a severe retinopathy and early-onset renal failure. British Journal of Ophthalmology 93:383-386.

21. Salviati L, Sacconi S, Murer L, Zacchello G, Franceschini L, Laverda AM, Basso G, Quinzii C, Angelini C, Hirano M, Naini AB, Navas P, DiMauro S, Montini G, Salviati L, Sacconi S, Murer L, Zacchello G, Franceschini L, Laverda AM (2005) Infantile encephalomyopathy and nephropathy with CoQ10 deficiency: a CoQ10-responsive condition. Neurology 65:606-608.
22. Park E, Ahn YH, Kang HG, Yoo KH, Won NH, Lee KB, Moon KC, Seong MW, Gwon TR, Park SS, Cheong HI (2017) COQ6 Mutations in Children With Steroid-Resistant Focal Segmental Glomerulosclerosis and Sensorineural Hearing Loss. American Journal of Kidney Diseases 70:139-144.
23. Korkmaz E, Lipska-Ziętkiewicz BS, Boyer O, Gribouval O, Fourrage C, Tabatabaei M, Schnaidt S, Gucer S, Kaymaz F, Arici M, Dinckan A, Mir S, Bayazit AK, Emre S, Balat A, Rees L, Shroff R, Bergmann C, Mourani C, Antignac C, Ozaltin F, Schaefer F, PodoNet C (2016) ADCK4-Associated Glomerulopathy Causes Adolescence-Onset FSGS. Journal of the American Society of Nephrology : JASN 27:63-68.
24. Lamont RE, Tan WH, Innes AM, Parboosingh JS, Schneidman-Duhovny D, Rajkovic A, Pappas J, Altschwager P, DeWard S, Fulton A, Gray KJ, Krall M, Mehta L, Rodan LH, Saller DN, Jr., Steele D, Stein D, Yatsenko SA, Bernier FP, Slavotinek AM (2016) Expansion of phenotype and genotypic data in CRB2-related syndrome. European Journal of Human Genetics 24:1436-1444.
25. Sivley MD, Wallace EL, Warnock DG, Benjamin WJ (2018) Conjunctival lymphangiectasia associated with classic Fabry disease. British Journal of Ophthalmology 102:54-58.
26. Orssaud C, Dufier J, Germain D (2003) Ocular manifestations in Fabry disease: a survey of 32 hemizygous male patients. Ophthalmic Genetics 24:129-139.
27. Michaud L (2013) Vascular tortuosities of the upper eyelid: a new clinical finding in fabry patient screening. Journal of ophthalmology 2013:207573.
28. Karr Jr WJ (1959) Fabry's disease (angiokeratoma corporis diffusum universale). An unusual syndrome with multisystem involvement and unique skin manifestations. American journal of medicine 27:829-835.
29. Pompen AWM, Ruiter M, Wyers HJG (1947) Angiokeratoma corporis diffusum (universale) Fabry, as a sign of an unknown internal disease; two autopsy reports. Acta Medica Scandinavica Stockholm 128:234-255.
30. Sher NA, Letson RD, Desnick RJ (1979) The ocular manifestations in Fabry's disease. Archives of Ophthalmology 97:671-676.
31. Spaeth GL, Frost P (1965) Fabry's disease. Its ocular manifestations. Archives of Ophthalmology 74:760-769.
32. Zavoreo I, Jurasic MJ, Lisak M, Jadrijevic Tomas A, Zrinscak O, Basic Kes V (2019) Recurrent Atypical Optic Neuritis as the Leading Sign of Fabry Disease. Acta Clinica Croatica 58:550-555.
33. Abe H, Sakai T, Sawaguchi S, Hasegawa S, Takagi M, Yoshizawa T, Usui T, Horikawa Y (1992) Ischemic optic neuropathy in a female carrier with Fabry's disease. Ophthalmologica 205:83-88.
34. Kumagai K, Mitamura Y, Mizunoya S, Fujimoto N, Yamamoto S (2008) A case of anterior ischemic optic neuropathy associated with Fabry's disease. Japanese Journal of Ophthalmology 52:421-423.
35. Oto S, Kart H, Kadayifcilar S, Ozdemir N, Aydin P (1998) Retinal vein occlusion in a woman with heterozygous Fabry's disease. European Journal of Ophthalmology 8:265-267.
36. Sodi A, Bini A, Mignani R, Minuti B, Menchini U (2009) Subfoveal choroidal neovascularization in a patient with Fabry's disease. International Ophthalmology 29:435-437.
37. Li AS, Ingham JF, Lennon R (2020) Genetic Disorders of the Glomerular Filtration Barrier. Clin J Am Soc Nephrol.

38. Maselli RA, Ng JJ, Anderson JA, Cagney O, Arredondo J, Williams C, Wessel HB, Abdel-Hamid H, Wollmann RL (2009) Mutations in LAMB2 causing a severe form of synaptic congenital myasthenic syndrome. Journal of Medical Genetics 46:203-208.
39. Hasselbacher K, Wiggins RC, Matejas V, Hinkes BG, Mucha B, Hoskins BE, Ozaltin F, Nürnberg G, Becker C, Hangan D, Pohl M, Kuwertz-Bröking E, Griebel M, Schumacher V, Royer-Pokora B, Bakkaloglu A, Nürnberg P, Zenker M, Hildebrandt F (2006) Recessive missense mutations in LAMB2 expand the clinical spectrum of LAMB2-associated disorders. Kidney International 70:1008-1012.
40. Bredrup C, Matejas V, Barrow M, Blahova K, Bockenhauer D, Fowler DJ, Gregson RM, Maruniak-Chudek I, Medeira A, Mendonca EL, Kagan M, Koenig J, Krastel H, Kroes HY, Saggar A, Sawyer T, Schittkowski M, Swietlinski J, Thompson D, VanDeVoorde RG, Wittebol-Post D, Woodruff G, Zurowska A, Hennekam RC, Zenker M, Russell-Eggitt I (2008) Ophthalmological aspects of Pierson syndrome. American Journal of Ophthalmology 146:602-611.
41. Falix FA, Bennebroek CA, van der Zwaag B, Lapid-Gortzak R, Florquin S, Oosterveld MJ (2017) A novel mutation of laminin beta2 (LAMB2) in two siblings with renal failure. European Journal of Pediatrics 176:515-519.
42. Mohney BG, Pulido JS, Lindor NM, Hogan MC, Consugar MB, Peters J, Pankratz VS, Nasr SH, Smith SJ, Gloor J, Kubly V, Spencer D, Nielson R, Puffenberger EG, Strauss KA, Morton DH, Eldahdah L, Harris PC (2011) A novel mutation of LAMB2 in a multigenerational mennonite family reveals a new phenotypic variant of Pierson syndrome. Ophthalmology 118:1137-1144.
43. Kagan M, Cohen AH, Matejas V, Vlangos C, Zenker M (2008) A milder variant of Pierson syndrome. Pediatric Nephrology 23:323-327.
44. Zenker M, Tralau T, Lennert T, Pitz S, Mark K, Madlon H, Dötsch J, Reis A, Müntefering H, Neumann LM (2004) Congenital nephrosis, mesangial sclerosis, and distinct eye abnormalities with microcoria: an autosomal recessive syndrome. American journal of medical genetics Part A 130A:138-145.
45. Wuhl E, Kogan J, Zurowska A, Matejas V, Vandevoorde RG, Aigner T, Wendler O, Lesniewska I, Bouvier R, Reis A, Weis J, Cochat P, Zenker M (2007) Neurodevelopmental deficits in Pierson (microcoria-congenital nephrosis) syndrome. American Journal of Medical Genetics Part A 143:311-319.
46. Cogan DG, Kruth HS, Datilis MB, Martin N (1992) Corneal opacity in LCAT disease. Cornea 11:595-599.
47. Fenske HD, Spitalny LA (1970) Hereditary Osteo-Onychodysplasia. American Journal of Ophthalmology 70:604-608.
48. Lichter PR, Richards JE, Downs CA, Stringham HM, Boehnke M, Farley FA (1997) Cosegregation of open-angle glaucoma and the nail-patella syndrome. Am J Ophthalmol 124:506-515.
49. Milla E, Hernan I, Gamundi MJ, Martinez-Gimeno M, Carballo M (2007) Novel LMX1B mutation in familial nail-patella syndrome with variable expression of open angle glaucoma. Molecular Vision 13:639-648.
50. Sweeney E, Fryer A, Mountford R, Green A, McIntosh I (2003) Nail patella syndrome: a review of the phenotype aided by developmental biology. Journal of Medical Genetics 40:153-162.
51. Bongers EM, Huysmans FT, Levtchenko E, de Rooy JW, Blickman JG, Admiraal RJ, Huygen PL, Cruysberg JR, Toolens PA, Prins JB, Krabbe PF, Borm GF, Schoots J, van Bokhoven H, van Remortele AM, Hoefsloot LH, van Kampen A, Knoers NV (2005) Genotype-phenotype studies in nail-patella syndrome show that LMX1B mutation location is involved in the risk of developing nephropathy. European Journal of Human Genetics 13:935-946.
52. Marini M, Bocciardi R, Gimelli S, Di Duca M, Divizia MT, Baban A, Gaspar H, Mammi I, Garavelli L, Cerone R, Emma F, Bedeschi MF, Tenconi R, Sensi A, Salmaggi A, Bengala M, Mari

F, Colussi G, Szczaluba K, Antonarakis SE, Seri M, Lerone M, Ravazzolo R (2010) A spectrum of LMX1B mutations in Nail-Patella syndrome: new point mutations, deletion, and evidence of mosaicism in unaffected parents. Genetics in Medicine 12:431-439.

53. Sekine T, Konno M, Sasaki S, Moritani S, Miura T, Wong WS, Nishio H, Nishiguchi T, Ohuchi MY, Tsuchiya S, Matsuyama T, Kanegane H, Ida K, Miura K, Harita Y, Hattori M, Horita S, Igarashi T, Saito H, Kunishima S (2010) Patients with Epstein-Fechtner syndromes owing to MYH9 R702 mutations develop progressive proteinuric renal disease. Kidney Int 78:207-214.

54. Han KH, Lee H, Kang HG, Moon KC, Lee JH, Park YS, Ha IS, Ahn HS, Choi Y, Cheong HI (2011) Renal manifestations of patients with MYH9-related disorders. Pediatr Nephrol 26:549-555.

55. Tabibzadeh N, Fleury D, Labatut D, Bridoux F, Lionet A, Jourde-Chiche N, Vrtovsnik F, Schlegel N, Vanhille P (2019) MYH9-related disorders display heterogeneous kidney involvement and outcome. Clin Kidney J 12:494-502.

56. Peterson L, Rao K, Crosson J, White J (1985) Fechtner syndrome--a variant of Alport's syndrome with leukocyte inclusions and macrothrombocytopenia. Blood 65:397-406.

57. Wang Z, Huang C, Sun Y, Lv H, Zhang M, Li X (2019) Novel mutations associated with autosomal-dominant congenital cataract identified in Chinese families. Experimental & Therapeutic Medicine 18:2701-2710.

58. Pecci A, Klersy C, Gresele P, Lee KJD, De Rocco D, Bozzi V, Russo G, Heller PG, Loffredo G, Ballmaier M, Fabris F, Beggiato E, Kahr WHA, Pujol-Moix N, Platokouki H, Van Geet C, Noris P, Yerram P, Hermans C, Gerber B, Economou M, De Groot M, Zieger B, De Candia E, Fraticelli V, Kersseboom R, Piccoli GB, Zimmermann S, Fierro T, Glembotsky AC, Vianello F, Zaninetti C, Nicchia E, Güthner C, Baronci C, Seri M, Knight PJ, Balduini CL, Savoia A (2014) MYH9-related disease: a novel prognostic model to predict the clinical evolution of the disease based on genotype-phenotype correlations. Human mutation 35:236-247.

59. Sanders FWB, Thompson E, Roberts H, Gupta N (2019) The use of pan-retinal photocoagulation to treat recurrent vitreous haemorrhage with neovascularisation in the context of Epstein syndrome: an MYH9-related disorder. BMJ Case Reports 12:29.

60. Fujita A, Tsukaguchi H, Koshimizu E, Nakazato H, Itoh K, Kuraoka S, Komohara Y, Shiina M, Nakamura S, Kitajima M, Tsurusaki Y, Miyatake S, Ogata K, Iijima K, Matsumoto N, Miyake N (2018) Homozygous splicing mutation in NUP133 causes Galloway–Mowat syndrome. Annals of Neurology 84:814-828.

61. Boutin TS, Charteris DG, Chandra A, Campbell S, Hayward C, Campbell A, Eye UKB, Vision C, Nandakumar P, Hinds D, andMe Research T, Mitry D, Vitart V (2020) Insights into the genetic basis of retinal detachment. Human Molecular Genetics 29:689-702.

62. Rosenberg AZ, Kopp JB (2017) Focal Segmental Glomerulosclerosis. Clin J Am Soc Nephrol 12:502-517.

63. Balreira A, Gaspar P, Caiola D, Chaves J, Beirão I, Lima JL, Azevedo JE, Miranda MCS (2008) A nonsense mutation in the LIMP-2 gene associated with progressive myoclonic epilepsy and nephrotic syndrome. Human Molecular Genetics 17:2238-2243.

64. Winn MP, Conlon PJ, Lynn KL, Farrington MK, Creazzo T, Hawkins AF, Daskalakis N, Kwan SY, Ebersviller S, Burchette JL, Pericak-Vance MA, Howell DN, Vance JM, Rosenberg PB (2005) A mutation in the TRPC6 cation channel causes familial focal segmental glomerulosclerosis. Science 308:1801-1804.

65. Reiser J, Polu KR, Moller CC, Kenlan P, Altintas MM, Wei C, Faul C, Herbert S, Villegas I, Avila-Casado C, McGee M, Sugimoto H, Brown D, Kalluri R, Mundel P, Smith PL, Clapham DE, Pollak MR (2005) TRPC6 is a glomerular slit diaphragm-associated channel required for normal renal function. Nat Genet 37:739-744.

66. Santin S, Ars E, Rossetti S, Salido E, Silva I, Garcia-Maset R, Gimenez I, Ruiz P, Mendizabal S, Luciano Nieto J, Pena A, Camacho JA, Fraga G, Cobo MA, Bernis C, Ortiz A, de Pablos AL, Sanchez-Moreno A, Pintos G, Mirapeix E, Fernandez-Llama P, Ballarin J, Torra R, Group FS, Zamora I, Lopez-Hellin J, Madrid A, Ventura C, Vilalta R, Espinosa L, Garcia C, Melgosa M,

Navarro M, Gimenez A, Cots JV, Alexandra S, Caramelo C, Egido J, San Jose MD, de la Cerda F, Sala P, Raspall F, Vila A, Daza AM, Vazquez M, Ecija JL, Espinosa M, Justa ML, Poveda R, Aparicio C, Rosell J, Muley R, Montenegro J, Gonzalez D, Hidalgo E, de Frutos DB, Trillo E, Gracia S, de los Rios FJ (2009) TRPC6 mutational analysis in a large cohort of patients with focal segmental glomerulosclerosis. Nephrol Dial Transplant 24:3089-3096.
67. Buscher AK, Konrad M, Nagel M, Witzke O, Kribben A, Hoyer PF, Weber S (2012) Mutations in podocyte genes are a rare cause of primary FSGS associated with ESRD in adult patients. Clin Nephrol 78:47-53.
68. Ogino D, Hashimoto T, Hattori M, Sugawara N, Akioka Y, Tamiya G, Makino S, Toyota K, Mitsui T, Hayasaka K (2016) Analysis of the genes responsible for steroid-resistant nephrotic syndrome and/or focal segmental glomerulosclerosis in Japanese patients by whole-exome sequencing analysis. J Hum Genet 61:137-141.
69. Roos RA, Maaswinkel-Mooy PD, vd Loo EM, Kanhai HH (1987) Congenital microcephaly, infantile spasms, psychomotor retardation, and nephrotic syndrome in two sibs. Eur J Pediatr 146:532-536.
70. Hou JW, Wang TR (1995) Galloway-Mowat syndrome in Taiwan. Am J Med Genet 58:245-248.
71. Colin E, Huynh Cong E, Mollet G, Guichet A, Gribouval O, Arrondel C, Boyer O, Daniel L, Gubler MC, Ekinci Z, Tsimaratos M, Chabrol B, Boddaert N, Verloes A, Chevrollier A, Gueguen N, Desquiret-Dumas V, Ferre M, Procaccio V, Richard L, Funalot B, Moncla A, Bonneau D, Antignac C (2014) Loss-of-function mutations in WDR73 are responsible for microcephaly and steroid-resistant nephrotic syndrome: Galloway-Mowat syndrome. Am J Hum Genet 95:637-648.
72. Vodopiutz J, Seidl R, Prayer D, Khan MI, Mayr JA, Streubel B, Steiß J-O, Hahn A, Csaicsich D, Castro C, Assoum M, Müller T, Wieczorek D, Mancini GMS, Sadowski CE, Lévy N, Mégarbané A, Godbole K, Schanze D, Hildebrandt F, Delague V, Janecke AR, Zenker M (2015) WDR73 Mutations Cause Infantile Neurodegeneration and Variable Glomerular Kidney Disease. Human Mutation 36:1021-1028.
73. Jinks RN, Puffenberger EG, Baple E, Harding B, Crino P, Fogo AB, Wenger O, Xin B, Koehler AE, McGlincy MH, Provencher MM, Smith JD, Tran L, Al Turki S, Chioza BA, Cross H, Harlalka GV, Hurles ME, Maroofian R, Heaps AD, Morton MC, Stempak L, Hildebrandt F, Sadowski CE, Zaritsky J, Campellone K, Morton DH, Wang H, Crosby A, Strauss KA (2015) Recessive nephrocerebellar syndrome on the Galloway-Mowat syndrome spectrum is caused by homozygous protein-truncating mutations of WDR73. Brain 138:2173-2190.
74. Jiang C, Gai N, Zou Y, Zheng Y, Ma R, Wei X, Liang D, Wu L (2017) WDR73 missense mutation causes infantile onset intellectual disability and cerebellar hypoplasia in a consanguineous family. Clinica Chimica Acta 464:24-29.
75. Jinks RN, Puffenberger EG, Baple E, Harding B, Crino P, Fogo AB, Wenger O, Xin B, Koehler AE, McGlincy MH, Provencher MM, Smith JD, Tran L, Al Turki S, Chioza BA, Cross H, Harlalka GV, Hurles ME, Maroofian R, Heaps AD, Morton MC, Stempak L, Hildebrandt F, Sadowski CE, Zaritsky J, Campellone K, Morton DH, Wang H, Crosby A, Strauss KA (2015) Recessive nephrocerebellar syndrome on the Galloway-Mowat syndrome spectrum is caused by homozygous protein-truncating mutations of WDR73. Brain 138:2173-2190.
76. Al-Rakan MA, Abothnain MD, Alrifai MT, Alfadhel M (2018) Extending the ophthalmological phenotype of Galloway-Mowat syndrome with distinct retinal dysfunction: a report and review of ocular findings. BMC Ophthalmology 18:147.
77. Ben-Omran T, Fahiminiya S, Sorfazlian N, Almuriekhi M, Nawaz Z, Nadaf J, Abu Khadija K, Zaineddin S, Kamel H, Majewski J, Tropepe V (2015) Nonsense mutation in the WDR73 gene is associated with Galloway-Mowat syndrome. Journal of Medical Genetics 52:381-390.

78. Bremond-Gignac D, Gerard-Blanluet M, Copin H, Bitoun P, Baumann C, Crolla JA, Benzacken B, Verloes A (2005) Three patients with hallucal polydactyly and WAGR syndrome, including discordant expression of Wilms tumor in MZ twins. American Journal of Medical Genetics Part A 134:422-425.
79. Lennon PA, Scott DA, Lonsdorf D, Wargowski DS, Kirkpatrick S, Patel A, Cheung SW (2006) WAGR(O?) syndrome and congenital ptosis caused by an unbalanced t(11;15)(p13;p11.2)dn demonstrating a 7 megabase deletion by FISH. American Journal of Medical Genetics, Part A 140:1214-1218.
80. Scott DA, Cooper ML, Stankiewicz P, Patel A, Potocki L, Cheung SW (2005) Congenital diaphragmatic hernia in WAGR syndrome. American Journal of Medical Genetics Part A 134:430-433.
81. Miller RW, Fraumeni JF, Manning MD (1964) Association of Wilms's Tumor with Aniridia, Hemihypertrophy and Other Congenital Malformations. New England Journal of Medicine 270:922-927.
82. Jadresic L, Leake J, Gordon I, Dillon MJ, Grant DB, Pritchard J, Risdon RA, Barratt TM (1990) Clinicopathologic review of twelve children with nephropathy, Wilms tumor, and genital abnormalities (Drash syndrome). Elsevier B.V., p 717.
83. Gronskov K, Olsen JH, Sand A, Pedersen W, Carlsen N, Bak Jylling AM, Lyngbye T, Brondum-Nielsen K, Rosenberg T (2001) Population-based risk estimates of Wilms tumor in sporadic aniridia. A comprehensive mutation screening procedure of PAX6 identifies 80% of mutations in aniridia. Human Genetics 109:11-18.
84. Crolla JA, van Heyningen V (2002) Frequent chromosome aberrations revealed by molecular cytogenetic studies in patients with aniridia. American Journal of Human Genetics 71:1138-1149.
85. Souza PVS, Badia BML, Silva LHL, Teixeira CAC, Seneor DD, Marin VDGB, Farias IB, Dias RB, Oliveira ASB, Pinto WBVR (2018) Leukodystrophy with disorders of sex development due to WT1 mutations. Journal of the Neurological Sciences 390:94-98.
86. Malfatti E, Laforet P, Jardel C, Stojkovic T, Behin A, Eymard B, Lombes A, Benmalek A, Becane HM, Berber N, Meune C, Duboc D, Wahbi K (2013) High risk of severe cardiac adverse events in patients with mitochondrial m.3243A>G mutation. Neurology 80:100-105.
87. Kurogouchi F, Oguchi T, Mawatari E, Yamaura S, Hora K, Takei M, Sekijima Y, Ikeda Si, Kiyosawa K (1998) A case of mitochondrial cytopathy with a typical point mutation for MELAS, presenting with severe focal-segmental glomerulosclerosis as main clinical manifestation. American journal of nephrology 18:551-556.
88. Doleris LM, Hill GS, Chedin P, Nochy D, Bellanne-Chantelot C, Hanslik T, Bedrossian J, Caillat-Zucman S, Cahen-Varsaux J, Bariety J (2000) Focal segmental glomerulosclerosis associated with mitochondrial cytopathy. Kidney International 58:1851-1858.
89. Hotta O, Inoue CN, Miyabayashi S, Furuta T, Takeuchi A, Taguma Y (2001) Clinical and pathologic features of focal segmental glomerulosclerosis with mitochondrial tRNALeu(UUR) gene mutation. Kidney International 59:1236-1243.
90. Guery B, Choukroun G, Noel LH, Clavel P, Rotig A, Lebon S, Rustin P, Bellane-Chantelot C, Mougenot B, Grunfeld JP, Chauveau D (2003) The spectrum of systemic involvement in adults presenting with renal lesion and mitochondrial tRNA(Leu) gene mutation. Journal of the American Society of Nephrology 14:2099-2108.
91. Lowik MM, Hol FA, Steenbergen EJ, Wetzels JF, van den Heuvel LP (2005) Mitochondrial tRNALeu(UUR) mutation in a patient with steroid-resistant nephrotic syndrome and focal segmental glomerulosclerosis. Nephrology Dialysis Transplantation 20:336-341.
92. Hirano M, Ricci E, Koenigsberger MR, Defendini R, Pavlakis SG, DeVivo DC, DiMauro S, Rowland LP (1992) Melas: an original case and clinical criteria for diagnosis. Neuromuscul Disord 2:125-135.

93. Kubota Y, Ishii T, Sugihara H, Goto Y, Mizoguchi M (1999) Skin manifestations of a patient with mitochondrial encephalomyopathy with lactic acidosis and strokelike episodes (MELAS syndrome). J Am Acad Dermatol 41:469-473.
94. Kaufmann P, Pascual JM, Anziska Y, Gooch CL, Engelstad K, Jhung S, DiMauro S, De Vivo DC (2006) Nerve conduction abnormalities in patients with MELAS and the A3243G mutation. Arch Neurol 63:746-748.
95. Yatsuga S, Povalko N, Nishioka J, Katayama K, Kakimoto N, Matsuishi T, Kakuma T, Koga Y (2012) MELAS: a nationwide prospective cohort study of 96 patients in Japan. Biochim Biophys Acta 1820:619-624.
96. Anglin RE, Garside SL, Tarnopolsky MA, Mazurek MF, Rosebush PI (2012) The psychiatric manifestations of mitochondrial disorders: a case and review of the literature. J Clin Psychiatry 73:506-512.
97. Alston CL, Bender A, Hargreaves IP, Mundy H, Deshpande C, Klopstock T, McFarland R, Horvath R, Taylor RW (2010) The pathogenic m.3243A>T mitochondrial DNA mutation is associated with a variable neurological phenotype. Neuromuscular Disorders 20:403-406.
98. Hwang JM, Park HW, Kim SJ (1997) Optic neuropathy associated with mitochondrial tRNA(Leu(UUR)) A3243G mutation. Ophthalmic Genetics 18:101-105.
99. Mariotti C, Savarese N, Suomalainen A, Rimoldi M, Comi G, Prelle A, Antozzi C, Servidei S, Jarre L, DiDonato S, Zeviani M (1995) Genotype to phenotype correlations in mitochondrial encephalomyopathies associated with the A3243G mutation of mitochondrial DNA. Journal of Neurology 242:304-312.
100. Hansrote S, Croul S, Selak M, Kalman B, Schwartzman RJ (2002) External ophthalmoplegia with severe progressive multiorgan involvement associated with the mtDNA A3243G mutation. Journal of the Neurological Sciences 197:63-67.
101. Luigetti M, Sauchelli D, Primiano G, Cuccagna C, Bernardo D, Lo Monaco M, Servidei S (2016) Peripheral neuropathy is a common manifestation of mitochondrial diseases: A single-centre experience. European Journal of Neurology 23:1020-1027.
102. Harrison TJ, Boles RG, Johnson DR, LeBlond C, Wong LJC (1997) Macular pattern retinal dystrophy, adult-onset diabetes, and deafness: A family study of A3243G mitochondrial heteroplasmy. American Journal of Ophthalmology 124:217-221.
103. Smith PR, Bain SC, Good PA, Hattersley AT, Barnett AH, Gibson JM, Dodson PM (1999) Pigmentary retinal dystrophy and the syndrome of maternally inherited diabetes and deafness caused by the mitochondrial DNA 3243 tRNA(Leu) A to G mutation. Ophthalmology 106:1101-1108.
104. Michaelides M, Jenkins SA, Bamiou DE, Sweeney MG, Davis MB, Luxon L, Bird AC, Rath PP (2008) Macular dystrophy associated with the A3243G mitochondrial DNA mutation: Distinct retinal and associated features, disease variability, and characterization of asymptomatic family members. Archives of Ophthalmology 126:320-328.
105. Rath PP, Jenkins S, Michaelides M, Smith A, Sweeney MG, Davis MB, Fitzke FW, Bird AC (2008) Characterisation of the macular dystrophy in patients with the A3243G mitochondrial DNA point mutation with fundus autofluorescence. British Journal of Ophthalmology 92:623-629.
106. Sivaprasad S, Kung BT, Robson AG, Black G, Webster AR, Bird A, Egan C (2008) A new phenotype of macular dystrophy associated with a mitochondrial A3243G mutation. Clinical & Experimental Ophthalmology 36:92-93.
107. Blum S, Robertson T, Klingberg S, Henderson RD, McCombe P (2011) Atypical clinical presentations of the A3243G mutation, usually associated with MELAS. Internal Medicine Journal 41:199-202.
108. de Laat P, Smeitink JAM, Janssen MCH, Keunen JEE, Boon CJF (2013) Mitochondrial retinal dystrophy associated with the m.3243A>G mutation. Ophthalmology 120:2684-2696.

109. Cao XY, Wei RB, Wang YD, Zhang XG, Tang L, Chen XM (2013) Focal segmental glomerulosclerosis associated with maternally inherited diabetes and deafness: clinical pathological analysis. Indian J Pathol Microbiol 56:272-275.
110. Guillausseau PJ, Massin P, Dubois-LaForgue D, Timsit J, Virally M, Gin H, Bertin E, Blickle JF, Bouhanick B, Cahen J, Caillat-Zucman S, Charpentier G, Chedin P, Derrien C, Ducluzeau PH, Grimaldi A, Guerci B, Kaloustian E, Murat A, Olivier F, Paques M, Paquis-Flucklinger V, Porokhov B, Samuel-Lajeunesse J, Vialettes B (2001) Maternally inherited diabetes and deafness: a multicenter study. Ann Intern Med 134:721-728.
111. Finsterer J, Scorza FA (2017) Renal manifestations of primary mitochondrial disorders. Biomed Rep 6:487-494.
